# Effect of Stimulus-Dependent Spike Timing on Population Coding of Sound Location in the Owl’s Auditory Midbrain

**DOI:** 10.1523/ENEURO.0244-19.2020

**Published:** 2020-04-07

**Authors:** M. V. Beckert, B. J. Fischer, J. L. Pena

**Affiliations:** 1 Dominick P. Purpura Department of Neuroscience, Albert Einstein College of Medicine, Bronx, NY 10461; 2 Department of Mathematics, Seattle University, Seattle, WA 98122

**Keywords:** barn owl, population coding, sound localization, spectrotemporal, synchrony

## Abstract

In the auditory system, the spectrotemporal structure of acoustic signals determines the temporal pattern of spikes. Here, we investigated this effect in neurons of the barn owl’s auditory midbrain (*Tyto furcata*) that are selective for auditory space and whether it can influence the coding of sound direction. We found that in the nucleus where neurons first become selective to combinations of sound localization cues, reproducibility of spike trains across repeated trials of identical sounds, a metric of across-trial temporal fidelity of spiking patterns evoked by a stimulus, was maximal at the sound direction that elicited the highest firing rate. We then tested the hypothesis that this stimulus-dependent patterning resulted in rate co-modulation of cells with similar frequency and spatial selectivity, driving stimulus-dependent synchrony of population responses. Tetrodes were used to simultaneously record multiple nearby units in the optic tectum (OT), where auditory space is topographically represented. While spiking of neurons in OT showed lower reproducibility across trials compared with upstream nuclei, spike-time synchrony between nearby OT neurons was highest for sounds at their preferred direction. A model of the midbrain circuit explained the relationship between stimulus-dependent reproducibility and synchrony, and demonstrated that this effect can improve the decoding of sound location from the OT output. Thus, stimulus-dependent spiking patterns in the auditory midbrain can have an effect on spatial coding. This study reports a functional connection between spike patterning elicited by spectrotemporal features of a sound and the coding of its location.

## Significance Statement

Spike timing of auditory neurons is modulated by the spectrotemporal structure of sounds. Whether this effect may have implications for coding of sound location remains unresolved. This question was approached in neurons of the owl’s midbrain, where temporal spiking patterns are modulated by the sound’s envelope while firing rate is driven by sound direction. We found that temporal patterns were dependent on binaural cues, leading to stimulus-dependent synchrony of nearby cells. Theoretical analysis showed that stimulus-dependent temporal patterning predicts stimulus-dependent synchrony in nearby cells sharing input with similar spectrotemporal structure, which in turn can sharpen the downstream readout of sound direction. This work shows how stimulus-dependent spike timing can affect the downstream coding of sound location by firing rate, a mechanism that can be generalized to sensory neurons sensitive to the temporal structure of the stimulus.

## Introduction

The temporal pattern of spiking of neurons in the auditory system is determined by the phase and amplitude modulations of frequency components of sound, known as “fine-structure” and “envelope,” respectively (Louage et al., 2004). The temporal precision of patterned responses varies along the auditory pathway, as downstream neurons display reduced capacity of locking to the phase of the stimulus fine structure (Johnson, 1980; Winter and Palmer, 1990; Köppl, 1997; Louage et al., 2004; Liu et al., 2006) and envelope (Lu et al., 2001; Joris et al., 2004; Christianson and Peña, 2006, 2007; Steinberg and Peña, 2011). These changes can result from the increased dimensionality of the neurons’ spectrotemporal tuning (Woolley et al., 2009; Kim and Doupe, 2011; Atencio et al., 2012) and emergent selectivities, such as to sound direction (Yao et al., 2015a,b).

**Figure 1.**
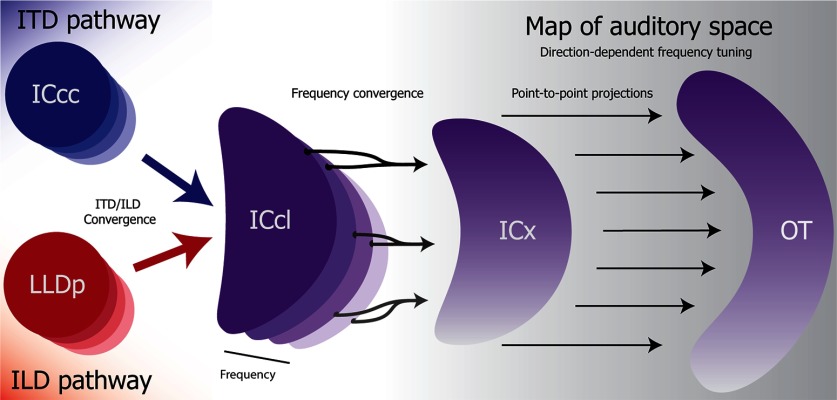
Schematic of the barn owl auditory midbrain. ITD (blue) and ILD (red) are computed along parallel brainstem pathways ending, respectively, in the core of the central nucleus of the inferior colliculus (ICcc) and the posterior part of the dorsal lateral lemniscus (LLDp). These regions are tonotopically organized (illustrated by contrast levels of color). The ITD and ILD pathways converge onto the lateral shell of the core of the inferior colliculus (ICcl). ICcl neurons display both ITD and ILD tuning (purple) and are also tonotopically organized. Frequency convergence onto the external nucleus of the inferior colliculus (ICx) contributes to the emergence of a map of auditory space. ICx sends point-to-point projections to the OT (Knudsen and Knudsen, 1983). Both ICx and OT display direction-dependent frequency tuning (illustrated with a purple gradient; Knudsen, 1984; Cazettes et al., 2014).

Like other animals, owls use disparities in arrival time (interaural time difference; ITD) and level (interaural level difference; ILD) of acoustic signals between the ears to localize sound (Knudsen et al., 1979; Moiseff, 1989). ITD and ILD are processed in parallel brainstem pathways (Takahashi et al., 1984) that converge in the lateral shell of the central nucleus of the inferior colliculus (ICcl), where neurons become tuned to both ITD and ILD (Takahashi et al., 1989; Adolphs, 1993; Fig. 1). Downstream from ICcl, a topographic representation of auditory space (referred to as space-map) emerges in the external nucleus of the inferior colliculus (ICx) and the optic tectum (OT; Knudsen and Konishi, 1978a,b; Knudsen, 1982). ICx neurons receive convergent input from ICcl (Wagner et al., 1987), which sharpens their spatial tuning through frequency convergence (Takahashi and Konishi, 1986; Mazer, 1998; Peña and Konishi, 2000).

**Figure 2.**
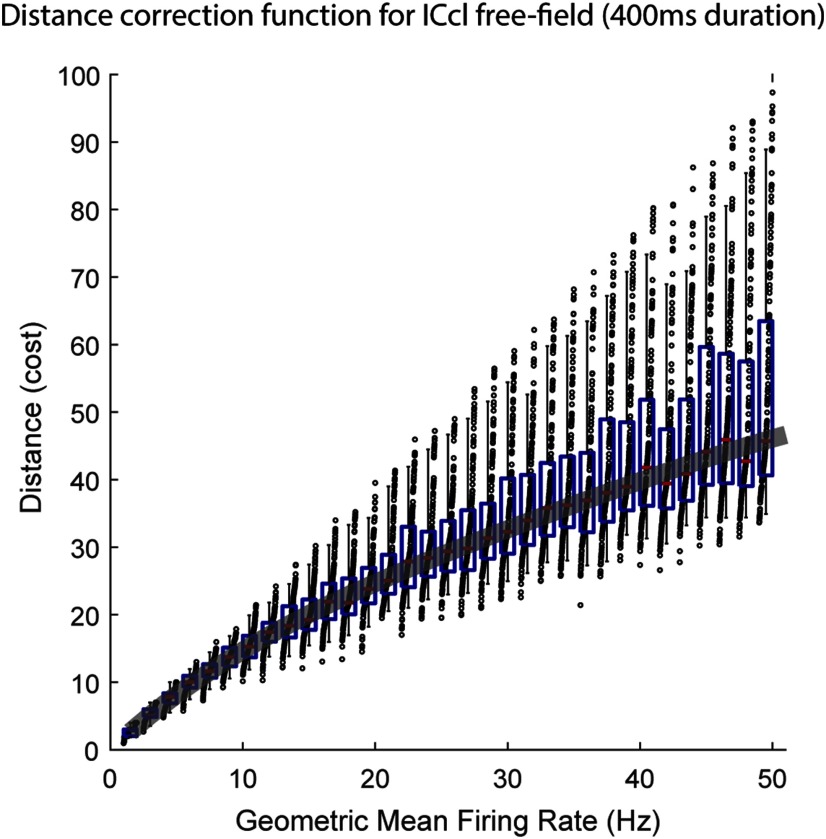
Effect of firing rate on spike-distance metric. Model spike trains with random spike timing were generated and their similarity measured using the spike-time distance metric described by (Victor and Purpura, 1996). GM firing rates were grouped in increments of 1.5 spikes per second. A fourth order polynomial was fit to the median values at varying joint firing rates. This function (thick black line) was then used to correct all data by the cost at chance levels. (boxplots: red line = median, blue box = first and third quartile, black whisker = 5% and 95%, dots are sorted data for each group.) A total of 20,000 simulations were run.

As in other species, temporal spiking patterns in the owl’s auditory brainstem pathways and midbrain are determined by the sound’s envelope (Keller and Takahashi, 2000; Steinberg and Peña, 2011; Steinberg et al., 2013). This patterning may facilitate the distinction between echoes and separate sources (Keller and Takahashi, 2000, 2005; Baxter et al., 2013). However, the bilateral matching of selectivity to spectrotemporal features of sounds in the inputs to ITD detector neurons (Fischer et al., 2011) and the dependence of selectivity to spectrotemporal features on ILD (Steinberg et al., 2013) in lower stages of the pathway suggests that this selectivity may depend on binaural cues. Whether this effect is conveyed to the ICcl is unknown.

Here, we built on previous work by investigating whether temporal spiking patterns may influence the coding of auditory space by midbrain neurons. We hypothesized that spike patterning induced by the sound’s envelope is affected by ITD and ILD, thus suggesting a functional relationship between selectivity to stimulus features linked to sound identity and location. We addressed this question by examining across-trial reproducibility of spike trains in ICcl elicited by identical copies of a sound, using standard metrics of spike-train distance (Victor and Purpura, 1996; Joris et al., 2006; Brette, 2012) as ITD, ILD, and sound direction in free field were varied. Reproducibility in ICcl was correlated with firing rate, as ITD and ILD delivered through earphones and sound direction in free field were independently manipulated, reaching a maximum at the preferred stimulus parameter in all cases.

Importantly, both ICx and OT display direction-dependent frequency selectivity (Knudsen, 1984; Cazettes et al., 2014; Fig. 1). Because the sound envelope is determined by the frequency spectrum (Louage et al., 2004), similar frequency selectivity in neurons with the same spatial tuning suggests these neurons may receive inputs with a similar envelope. In addition, it has recently been shown that nearby neurons in OT may share inputs (Beckert et al., 2017). However, neurons downstream the auditory pathway typically show reduced envelope locking (Lu et al., 2001; Joris et al., 2004; Louage et al., 2004; Christianson and Peña, 2007; Steinberg and Peña, 2011; Steinberg et al., 2013; Yao et al., 2015a,b). Thus, whether temporal spiking patterns in ICcl have an effect on downstream neurons is an open question. We tested the hypothesis that while single OT neurons would display reduced ability to lock to the envelope, an effect on temporal correlations remained at the population level. To test this, tetrodes were used to simultaneously record multiple single units in OT.

Consistent with our hypothesis, synchrony of neighboring OT neurons varied in a stimulus-dependent manner. Modeling explained the relationship between stimulus-dependent spiking patterns determined by the sound’s envelope and downstream synchrony, and that stimulus-dependent synchrony could affect the decoding of the OT population through disambiguating population responses. These results demonstrate an interaction between spike timing driven by spectrotemporal properties of sounds and coding of sound location that may have a role in behavioral performance.

## Materials and Methods

### Subjects and surgery

Adult American barn owls (*Tyto furcata)* of both sexes (four male, one female) were implanted with custom-built stainless steel headplates held in place with dental acrylic. A well was molded in the acrylic posterior of the headplate, aimed over ICcl and OT.

Owls were food-deprived at least 12 h before each procedure. Anesthesia was induced by intramuscular injections of ketamine (Ketaset; 20 mg/kg) and xylazine (Anased; 2 mg/kg). Owls were provided with prophylactic antibiotic (Ampicillin; 20 mg/kg, i.m.) and subcutaneous hydration (lactated Ringer’s solution; 10 ml, s.c.). A proper anesthesia level was assessed via pedal and eyelid reflex. This level of anesthesia was maintained with subsequent half-doses of ketamine and xylazine throughout the recording session. Owls were wrapped with a heating pad to maintain body temperature.

At the end of the recording session, the craniotomy was covered with a sterile plastic disk and the well was filled with a quick-dry silicone compound (Quick-Pro, Warner Tech-Care). An analgesic was administered to prevent pain after recovery from anesthesia (Rimadyl; 3 mg/kg, i.m.). Owls were allowed to recover in a small crate overnight. Once all signs of physical impairment were absent, they were returned to their home aviary. Subsequent recording sessions were always 10 or more days later to allow for proper recovery. All procedures were in compliance with guidelines set by the National Institute of Health and the Albert Einstein College of Medicine animal care committee’s regulations.

### Data collection

All recordings were performed in a double wall sound attenuating chamber (Industrial Acoustics) lined with anechoic acoustic foam (Sonex). ICcl and OT were targeted using known stereotaxic coordinates relative to the intersection of the midline and interaural line. Small openings in the dura were made with a sterile needle to facilitate electrode entry into the brain. Tungsten electrodes (5-MΩ resistance; A-M Systems) were driven with a microdrive (Newport) into ICcl. These high-impedance electrodes were able to isolate single units from the strong evoked field potentials of ICcl. Data acquisition was performed using a TDT system 3 (Tucker-Davis Technologies) and custom-written MATLAB routines (Tytology). A threshold was chosen for single cell isolation based on online visual monitoring of spike amplitudes.

Recordings in OT were performed using tetrodes (Q-trodes; NeuroNexus) driven with a Kopf microdrive (David Kopf Instruments). A Plexon Omniplex system (SortClient, Plexon) was used for data acquisition. Recordings were performed on sites containing at least two visually well-isolated units. Offline sorting software was used to confirm isolation (Offline Sorter, Plexon). On average, four to five units were recorded per site.

Electrode locations within both brain regions were confirmed using established physiological characteristics including tuning to ITD, ILD, and frequency in both ICcl (Takahashi and Konishi, 1986; Wagner et al., 1987; Takahashi et al., 1989; Bremen et al., 2007) and OT, as well as bursting discharge and response to light stimulation in OT (Knudsen, 1982, 1984).

Because of spike-sorting constraints, bursting responses, characteristic of superficial layers of OT, were excluded from the analysis. Therefore the OT units included in the analysis are likely from deep OT layers (Knudsen, 1982).

### Acoustic stimulation

#### Dichotic (earphone) stimulation

Acoustic stimulation was performed using previously described methods. In brief, TDT system three and custom MATLAB routines were used to synthesize and deliver all stimuli. Custom-made earphones containing a speaker (Knowles, model 1914) and a microphone (Knowles, model 1319) were inserted into the owl’s ear canal. Earphone speakers were calibrated using the earphone microphones each time they were positioned in the ear canals. Broadband sounds (0.5–10 kHz; duration, 150 ms; interstimulus interval, 300 ms) were presented randomly for either varying ITDs or ILDs to measure tuning functions. For ITD and ILD tuning characterization, broadband stimuli were not identical in each presentation (unfrozen noise). After ITD and ILD tuning was characterized, identical broadband stimuli (frozen noise) with a longer duration (450 ms) presented 100–300 times were used to assess spiking reproducibility (see Data Analysis section below). A new frozen noise token was generated for each neuron.

#### Free-field stimulation

Free-field sound stimulation was presented through a custom built semi-spherical array of speakers (Sennheiser, 3P127A) surrounding the stereotax. Speaker positions ranged from ±100° azimuth and ±80° elevation with spacing between 10° and 30°. Owls were positioned to face the 0° azimuth and 0° elevation speaker for all recordings. Speakers were calibrated using a Brüel and Kjær microphone (model 4190). Broadband signals (0.5–10 kHz) were transformed by the calibration filter for each speaker to equalize sounds across the array. Stimulus duration (150 ms) and interstimulus intervals (300 ms) were the same as those for dichotic stimulation. Speakers were activated randomly 20–40 times to measure a spatial receptive field (SpRF). Broadband noise stimuli used for SpRF characterization were not identical in each presentation (unfrozen noise). As for experiments using dichotic stimulation, after characterization of the SpRF, longer stimuli (250 ms) presented 100–300 times were used to assess reproducibility (see Data Analysis section below). A new frozen noise token was generated for each set of simultaneously recorded neurons.

### Data Analysis

#### Tuning curves

ITD, ILD, and spatial tuning curves were computed from the average number of spikes elicited during sound presentation over 10–30 repetitions of broadband noise. Neurons were classified as having a significant response to auditory stimulation if the average response to any stimulus condition was 2 SD larger than the mean spontaneous firing rate, which was measured by counting spikes preceding the stimulus, during an equivalent amount of time as the stimulus duration (all neurons fit this criterion).

#### Response reproducibility

Across-trial reproducibility of spike trains was calculated using a shuffled autocorrelogram (SAC) as previously reported (Joris et al., 2006; Brette, 2012). Neurons in ICcl and OT characteristically display a strong onset response (Knudsen, 1984). In order to ensure measurements of reproducibility were not biased by onset responses, the first 50 ms after stimulus onset were excluded. SACs are histograms of time intervals between each spike in a stimulus trial and every spike in other trials. Reproducibility was defined as the integral of the center SAC peak normalized by firing rate (*m*), to account for the effect of chance (Brette, 2012):

$$reproducibility=\frac{\int_{-window}^{+window}\left(SAC-m^{2}\right)dt}{m},$$

where SAC is the SAC, which is here integrated over a time range (± window) centered at 0 lag, after normalizing by subtracting the squared mean firing rate, and then dividing by the mean firing rate. SACs are used as a metric of reproducibility of firing patterns across trials (Joris et al., 2006; Brette, 2012). The window for integration is functionally equivalent to the coincidence window described in previous studies (Joris et al., 2004, 2006; Louage et al., 2004). Because the half-width of the SAC’s center peak can be considered a proxy for the temporal precision of a neuron’s spiking (Louage et al., 2004), we set the SAC’s integration window to the median half-width of the SAC’s center peak across neurons of each dataset. To determine this half-width, we adjusted the bin size until the mean SAC displayed a significant peak [SAC value at 0 lag was greater than 2 SD above average in the flanks (±20- to 50-ms lag)] for each dataset. All bin sizes tested (0.05–20 ms; Joris et al., 2006) displayed significant peaks for ICcl while OT required a bin size of at least 2 ms. Half-width was therefore calculated using 100-μs bin size for ICcl and 2 ms for OT (median half-width: ICcl dichotic = 1 ms, ICcl free-field = 1 ms, OT free-field = 10 ms). Python code for this analysis was provided by Romain Brette (Brette, 2012).

For ICcl, reproducibility and firing rate were assessed for 6–10 different ITD and ILD values for dichotic stimulation, or a similar number of speakers for free-field stimulation, including those inducing maximal responses and points along the dynamic range of tuning curves. For OT, reproducibility was measured across speakers in the entire free-field array.

#### Spike train distance

As a complementary metric to the SAC analysis, the patterning of responses was assessed using a spike-train distance calculation, simply referred to as distance. Distance calculations measure the cost of transforming one spike train into another as described by Victor and Purpura (1996). This metric uses three different elementary transformations to modify spike trains: (1) insert a spike, (2) remove a spike, or (3) time shift a spike. The cost of inserting or removing a spike is assigned a value of 1; while shifting a spike a given amount of time (Δ*t* seconds) is assigned a cost of *q*|Δ*t*|, where *q* is a unitless scaling factor inversely related to precision (Victor, 2005). We set *q* to a previously used value of 100 (Machens et al., 2001). Other values of *q*, 33 (Aronov et al., 2003), 64 (Victor and Purpura, 1996), 250 (Middlebrooks et al., 1994), were also tested and did not affect the general trends reported.

Permutations of all possible elementary transformations were performed using MATLAB code (code obtained from the laboratory website of Jonathan Victor at Cornell Medical School; http://www-users.med.cornell.edu/∼jdvicto/pubalgor.html), the solution which displayed the smallest final cost was then selected, and this cost was reported. Distance was calculated for the same data used for the SAC analysis. Here, we compared the distance between spike trains for each stimulus trial in a pairwise manner and averaged all values to obtain a final distance for each ITD, ILD, and free-field direction of each neuron.

The distance metric is zero for empty spike trains (Victor and Purpura, 1996). Thus, we omitted trials where neurons did not fire any spikes to prevent them from biasing results.

In addition, average distance increases with firing rate. To ensure that firing rate was not underlying stimulus-dependent changes in distance, we generated a correction function. Pairs of random spike trains were generated by selecting *n* spike times from a uniform distribution over intervals matching the stimulus durations used in our study. Distance as a function of firing rate was estimated by varying the value of *n*. To take into account the across-trial variability in firing rate observed in the data, disparities of as much as twice the firing rate differences between the two model trains were allowed. To select the function that best fit the relationship between mean firing rate and distance between model spike trains, two observations were considered. First, the data displayed a nonlinear relationship (Fig. 2), and therefore, a simple linear regression would not suffice. Additionally, because the distance between trials with zero spikes will be zero by construction of this metric, when fitting the function, the y-intercept was forced to zero (MATLAB code *polyfitZero* obtained from MATLAB Central, written by Mark Mikofski; https://www.mathworks.com/matlabcentral/fileexchange/35401-polyfitzero). A fourth order polynomial was necessary to ensure that residuals were distributed normally, indicating this function was tracking the data properly. The curve was fitted using the range of firing rates observed by the data (0–51.5 maximum spike count; Fig. 2). Because stimulus duration limits the distance metric and it varied across datasets (200 and 400 ms for dichotic and free field stimulation in ICcl, respectively, and 100 ms for OT, onset time omitted), the expected relationship between distance and spike count was estimated separately for a range of stimulus durations. The increase in distance with spike count predicted by chance was:

1. for a 100-ms stimulus:
  $$expected\;distance=\left(-1.19\text{x}\;10^{-5}\right)x^{4}+\left(1.30\text{x}\;10^{-3}\right)x^{3}-0.05x^{2}+1.38x$$
2. for a 200-ms stimulus:
  $$expected\;distance=\left(-1.03\text{x}\;10^{-5}\right)x^{4}+\left(1.30\text{x}\;10^{-3}\right)x^{3}-0.06x^{2}+1.70x$$
3. for a 400-ms stimulus:
  $$expected\;distance=\left(-5.62\text{x}\;10^{-6}\right)x^{4}+\left(7.74\text{x}\;10^{-4}\right)x^{3}-0.04x^{2}+1.82x,$$

where *x* is the geometric mean (GM) firing rate between the two spike trains (spikes/second). Distances for actual data were then corrected by subtracting the values expected by chance, given the corresponding stimulus duration. Thus, negative values indicate the spike trains are more similar than expected by chance.

Distance values displayed notable outliers (detected using median absolute deviation method*s*) which made the distribution of distances across the population heteroscedastic (variability was not constant across firing rate). Because Pearson correlation makes the assumption of homoscedasticity and that there are no outliers, we used Spearman rank-order correlation coefficients to evaluate the relationship between distance and firing rate.

#### Spike-time synchrony of space-map cells

Synchrony between simultaneously recorded OT neurons stimulated in free-field was assessed using previously described methods. In brief, cross-correlograms (CCGs) of pairs of simultaneously recorded neurons were computed for sound emitted by speakers in different locations. As for reproducibility and distance computations, spikes within the onset of the stimulus presentation (first 50 ms) were omitted from the analysis. Spikes within 1 ms of one another were deemed coincident. CCGs were smoothed using a 5-ms sliding window (Bair et al., 2001; Kohn and Smith, 2005). Adding up all coincidences yielded a standard-CCG. To determine whether reproducible firing across trials influenced this synchrony, a shifted-CCG (Bair et al., 2001) was also computed. The shifted-CCG measures the number of coincident spikes between two neurons across non-simultaneous trials of identical sounds, specifically counting coincidences from trial *n* of neuron 1 and trial *n  +  1* of neuron 2. This CCG captures the amount of coincidences that may be due to spiking patterns driven by temporal dynamics of the stimulus. Here, we used the shifted-CCG to investigate whether synchrony of nearby cells could be explained by patterned activity of these cells. To evaluate coincidences not related to temporal dynamics of the stimulus, the shifted-CCG was subtracted from the standard-CCG for each neuron (coincidences for each time lag bin in the shifted-CCG were removed from the matching bin in the standard-CCG), to produce a corrected-CCG. To take into account coincidences occurring due to chance alone related to firing rate and bin size, CCGs were normalized by the bin size multiplied by the GM firing rate (square root of the product of firing rates of the two neurons), as in other studies (Bair et al., 2001). Synchrony was quantified by the integral of the CCG’s center peak. A standard boundary was selected by averaging the CCG of all neurons and identifying a range which fit this large center peak. For this dataset the boundary was ±10 ms. Sound locations that did not elicit sufficient spiking (<2 SD above the mean baseline) for both neurons in a pair were omitted from the calculation of synchrony. In addition, pairs needed to display sufficient spiking for at least two speakers to be included in the dataset (189/194 pairs fit criterion).

To assess how synchrony varied with positions in space, GM was calculated for the response of pairs of neurons to each speaker location. The GM firing rate is a standard metric for assessing the combined firing rate of neurons (Bair et al., 2001; Kohn and Smith, 2005; Smith and Kohn, 2008).

#### Up-and-down states

To investigate whether up-and-down states induced by anesthesia underlay spiking co-variation, we used a method described by Pachitariu et al. (2015) to detect up-states based on spike counts. The spike times of all units recorded by a single tetrode were pooled to attain a measurement of multiunit activity (MUA) during baseline recordings, in the absence of sound stimulation. Spike times were binned in 10-ms intervals to create a running spike count. A 10-bin wide median filter (custom-written MATLAB routine, but see function *medfilt1*) was applied to the spike count, where each value was replaced by the median spike count of the 10 neighboring bins (edges were omitted). Up-and-down states are generally hundreds to thousands of milliseconds long (Parga and Abbott, 2007; McFarland et al., 2011; Bragin et al., 2012; Xu and Wang, 2014), therefore this filter window was sufficient to identify potential up-states. Bouts of firing across more than half of the bins of the median filter window (six or more in 10) yielded median values above 0, the criterion used by this method to define up-states.

#### Correlation analysis

The Pearson product-moment correlation coefficient (*r*) was calculated to describe the relationship between reproducibility and distance with mean firing rate of each neuron, and the same was done for synchrony and the GM firing rate for each pair of neurons. The r values were transformed using Fisher’s z-transformation for statistical tests and then converted back to *r*s for reporting. Normalized reproducibility and mean firing rates were also pooled and tested for overall correlation, and the same was done for synchrony and GM firing rate. The significance of the correlation was tested using a two-tailed *t* test.

### Computational model

We used a model to investigate how stimulus dependent spiking patterns in ICcl could affect synchrony between nearby neurons in OT. The input to ICcl was modeled using a previously described model of the auditory periphery in the barn owl (Fischer et al., 2009). Briefly, broadband Gaussian noise signals between 0.2 and 12 kHz were passed through a gammatone filter-bank with center frequencies ranging from 1 to 9 kHz in 0.2-kHz steps. The time constants of the filters were specific to the owl and estimated from Köppl (1997) to model cochlear filters. The outputs of the cochlear filters were cross-correlated in each frequency channel. The cross-correlation model included a gain-control component so that the amplitude of the cross-correlation output grows with stimulus level in a manner that matches the relationship between stimulus level and firing rate in barn owl coincidence detector neurons (Fischer et al., 2009).

ICcl neurons were modeled as adaptive exponential leaky integrate-and-fire neurons that were driven by the output of the cross-correlation model. The adaptive exponential leaky integrate-and-fire model has been shown to be a good model of intracellular responses in the barn owls’ midbrain (Fontaine et al., 2014b). The subthreshold membrane potential of the *i*th ICcl neuron obeyed the differential equation:

$$\tau \frac{dV_{i}\left(t\right)}{dt}=\left(V_{L}-V_{i}\left(t\right)\right)+\Delta \text{exp}\left(\frac{V_{i}\left(t\right)-\theta_{i}\left(t\right)}{\Delta }\right)+g_{i}\left(t\right)\left(V_{E}-V_{i}\left(t\right)\right),$$

where the membrane time constant *τ* = 60 ms, the leak reversal potential *V_L_* = –75 mV, the synaptic reversal potential *[dot]VE* = 0 mV, *θ_i_*(*t*) is the dynamic spiking threshold, Δ = 1 mV, and *g_i_*(*t*) is the input conductance. Note that the input conductance *g_i_*(*t*) is normalized by the leak conductance, and therefore *g_i_*(*t*) has no units. The input conductance is a function of the output of the cross-correlation model at the best ITD and best frequency of the *i*th ICcl neuron, which is denoted ξ(*t*) The cross-correlation output was transformed using an exponential nonlinearity followed by a linear rectification to generate the input signals to the modeled ICcl cells:

$$s_{i}\left(t\right)=a{\left[\exp\left(x_{i}\left(t\right)\right)-b_{i}\right]}_{+},$$

where *b_i_* is the average value of exp(ξ(*t*)) and *a *=* *45. The input conductance *g_i_*(*t*) = *s_i_*(*t*)  +  *n_i_*(*t*) is a sum of *s_i_*(*t*) and a neuron-specific noise signal *n_i_*(*t*) The noise *n_i_*(*t*) was a lowpass Gaussian noise signal with flat amplitude spectrum up to 1 kHz. The root mean square (RMS) level of the noise was proportional to the square root of the RMS level of the signal *s_i_*(*t*) so that the signal-to-noise ratio grows as the square root of the RMS level of the signal. A neuron-specific noise signal *n_i_*(*t*) was included to model differences in spike-train reproducibility between neurons, and therefore the noise signals were independent across neurons.

#### The dynamic spiking threshold obeyed the differential equation

$$\tau_{\theta }\frac{d\theta_{i}\left(t\right)}{dt}=V_{1}+k_{1}\ln(1+\exp\left(\frac{V_{i}\left(t\right)-V_{2}}{k_{2}}\right)),$$

where *τ_θ_* = 5 ms, *V*_1_ = –57 mV, *k*_1_ = 5 mV, *k*_2_ = 1 mV, and *V*_2_ = –67 mV. A spike occurs when the membrane potential exceeds the dynamic threshold *θ_i_*(*t*), after which the membrane potential is reset to –55 mV and held constant for a refractory period of 1 ms.

The ICcl spikes formed the input to a population of space-map neurons. The subthreshold membrane potential of the *i*th space-map neuron obeyed the differential equation:

$$\tau \frac{dV_{i}\left(t\right)}{dt}=\left(V_{L}-V_{i}\left(t\right)\right)+\Delta \exp\left(\frac{V_{i}\left(t\right)-\theta_{i}\left(t\right)}{\Delta }\right)+\overset{N}{\underset{j=1}{\sum }}\omega_{ij}g_{syn,j}\left(t\right)\left(V_{syn,j}-V_{i}\left(t\right)\right),$$

where *ω_ij_* is a connection weight, *g_syn,j_*(*t*) is the synaptic conductance due to the response of the *j*th input neuron, *N* = 2665 is the number of input ICcl neurons, and the synaptic reversal potential *V_syn,j_* is equal to 0 mV for excitatory synapses and –80 mV for inhibitory synapses. The parameters *τ*, *V_L_*, and Δ of the membrane potential and the parameters of the dynamic spiking threshold for the space-map neurons were the same as described for the ICcl neurons. The connection weights varied with the difference in the best ITDs and best frequencies of the ICcl and space-map neurons, *ω_ij_* ∝ ω*_ITD,ij_* × *ω_F,ij_*. The pattern of the ITD-dependent component of the weights was given by the difference between a central Gaussian centered at best ITD of the space map neuron and two Gaussians centered at ITDs that differed from the best ITD by one half of the period of the neuron’s best frequency:

$$\omega_{ITD,ij}=\;\text{exp}\left(-\frac{1}{2}{\left(\frac{ITD_{i}-ITD_{j}}{\sigma }\right)}^{2}\right)-\frac{1}{2}\exp\left(-\frac{1}{2}{\left(\frac{ITD_{i}+0.5P_{i}-ITD_{j}}{\sigma }\right)}^{2}\right)-\frac{1}{2}\exp\left(-\frac{1}{2}{\left(\frac{ITD_{i}-0.5P_{i}-ITD_{j}}{\sigma }\right)}^{2}\right),$$

where *ITD_i_* is the best ITD of the space-map neuron, *ITD_j_* is the best ITD of the jth ICcl neuron, *P_i_* is the period of the space-map neuron’s best frequency, and *σ* = 10 μs. The pattern of the frequency-dependent component of the weights was given by a Gaussian centered at best frequency of the space map neuron $\omega_{F,ij}=\;\text{exp}\left(-\frac{1}{2}{\left(\frac{BF_{i}-BF_{j}}{\sigma }\right)}^{2}\right)$, where *BF_i_* is the best frequency of the space-map neuron, *BF_j_* is the best frequency of the jth ICcl neuron, and *σ* = 6500–20 × *ITD_i_* Hz. The connection weight *ω_ij_* was the magnitude of the product of these values, while its sign determined whether the input was excitatory or inhibitory. For excitatory inputs the synaptic reversal potential was *V_syn,j_* = 0 mV and for inhibitory inputs the synaptic reversal potential was *V_syn,j_* = 80 mV. The synaptic conductance was an exponential: $g_{syn,j}\left(t\right)=\underset{n}{\sum }\exp\left(-\frac{t-t_{j,n}}{\tau_{s}}\right)H\left(t-t_{j,n}\right)$, where *t_j,n_*is the time of the *n*th spike of the *j*th ICcl neuron and *H*(t) is the unit step function. The time constant of the synaptic conductance was *τ_s_* = 5 ms.

We modeled a population of neurons downstream from the auditory space map to investigate the effects of spiking synchrony in the space map on the coding of ITD. The subthreshold membrane potential of the *i*th output neuron was modeled using the same form of model as used for the space map:

$$\tau \frac{dV_{i}\left(t\right)}{dt}=\left(V_{L}-V_{i}\left(t\right)\right)+\Delta \exp\left(\frac{V_{i}\left(t\right)-\theta_{i}\left(t\right)}{\Delta }\right)+\overset{N}{\underset{j=1}{\sum }}\omega_{ij}g_{syn,j}\left(t\right)\left(V_{syn,j}-V_{i}\left(t\right)\right),$$

but here *g_syn,j_*(*t*) is the synaptic conductance from the *j*th space map neuron and *N* = 81 is the number of input space-map neurons. The connection weights between neurons in the input and output layers of the model followed a Gaussian-shaped function with a SD of 30°. The SD accounted for the population in the input layer that converged onto the output layer. The results were robust to changes in the SD over the range 5–75°.

Synchrony in the modeled populations was quantified with the same types of CCGs used for space-map neurons (described in section Spike-time synchrony of space-map cells above). The effect of stimulus-dependent synchrony in the space-map population was assessed by the suppression of side peaks forming in the pattern of spike counts produced by the output population when plotted as a function of the preferred ITD. We first measured the side peaks in the output population for the original model data displaying stimulus-dependent synchrony. We next altered the synchrony in the side peak by shifting spike times of neurons in the side peak. To manipulate synchrony in side peaks by shifting spike times, the stimulus interval was first divided into 25-ms segments. The average spike time within side peaks was calculated for each 25-ms segment across modeled neurons, to create a template spike timing. In each 25-ms segment, spike times were shifted toward these template spike times. The amount of shift toward the mean time was controlled to produce synchrony in the side peak that matched the synchrony in the main peak. This manipulation did not change the overall firing rate of the neurons. We then compared the side peak in the output population generated from this new space-map population where synchrony is high in the side peak.

For calculating reproducibility and synchrony in modeled neurons, the first 50 ms of the onset was omitted to match the analysis of neural data.

### Statistical analysis

All datasets were tested for normalcy (D’Agostino–Pearson normality test) using GraphPad Prism 7. Non-parametric tests were used when at least one group was not normally distributed. Statistical tests used are listed in the results. Correlation coefficients were computed using MATLAB. Quantification of data are written as mean ± SD unless otherwise noted.

### Code accessibility

Relevant code for calculation of reproducibility, spike-time distance, spike-time synchrony, and model neuron populations are publicly available through GitHub at https://github.com/penalab/Beckert-et-al.-2020.

## Results

To assess the precision of spiking patterns for sounds from different locations, we measured across-trial reproducibility in single units recorded from the lateral shell of the central nucleus of the inferior colliculus (ICcl; *n* = 68 units). Reproducibility was quantified by computing the integral of the central peak of the SAC, a metric of the similarity and temporal precision of spike timing across trials of identical (frozen) broadband noise (Joris et al., 2006; Brette, 2012), and the across-trial spike distance (Victor and Purpura, 1996). Stimuli were presented either through earphones (varying binaural spatial cues) or in free-field (varying spatial location). The analysis of reproducibility in ICcl was followed by tetrode recordings in the downstream map-of-space in the OT (*n* = 93 units from 20 recording sites, resulting in 194 pairs of simultaneously recorded neurons). Across-trial reproducibility and distance, as well as spike-time synchrony, were also examined in OT neurons. Finally, the underlying network mechanisms and coding implications of these findings were assessed through computational modeling.

### Reproducibility in ICcl depends on binaural cues

**Figure 3.**
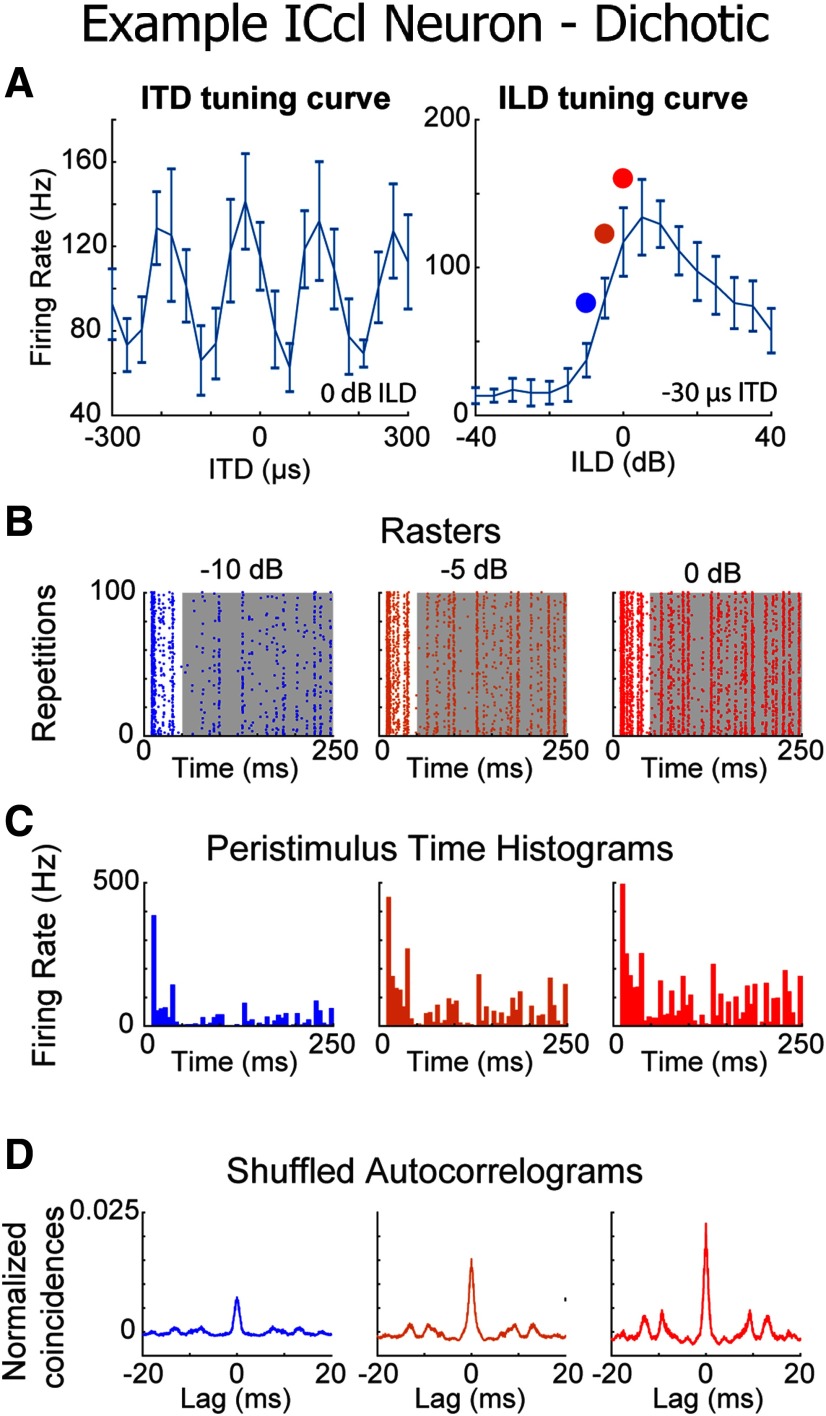
Reproducibility in ICcl to varying binaural cues. ***A***, Tuning curves of an example ICcl neuron for ITD (left) and ILD (right). Positive ITDs and ILDs indicate, respectively, sounds leading and higher intensity on the right ear (contralateral side for the example cell). Colored dots in the ILD tuning curve are the examples displayed in ***B–D*** (plots represent mean ± SEM, *n* = 10 repetitions per trial). ***B***, Example spiking raster plots of neuron shown in ***A*** at varying ILDs (indicated on top of each plot). Gray background represents region of stimulation used for SAC and distance analysis (50-ms onset omitted). ***C***, Peristimulus time histograms generated from the raster plots in ***B*** (5-ms bin size). ***D***, SACs generated from the rasters in ***B***.

Single units in ICcl displayed tuning to the primary binaural cues for sound localization, ITD and ILD, as previously described (Keller and Takahashi, 2000; Fischer et al., 2007; Singheiser et al., 2012). In particular, rate-ITD curves of ICcl neurons were quasi-periodic with a larger center peak but prominent side peaks, and rate-ILD curves displayed sigmoidal or open peak shapes favoring higher sound level on the contralateral side (Fig. 3*A*).

Neurons displayed reproducible firing across trials, resulting in patterned spike rasters (Fig. 3*B*), peaks in post-stimulus time histograms (Fig. 3*C*), and large peaks at 0 delay in the SACs (Fig. 3*D*). This is consistent with previous reports that ICcl neurons exhibit reproducible spike timing across trials of identical sounds (Keller and Takahashi, 2000).

**Figure 4.**
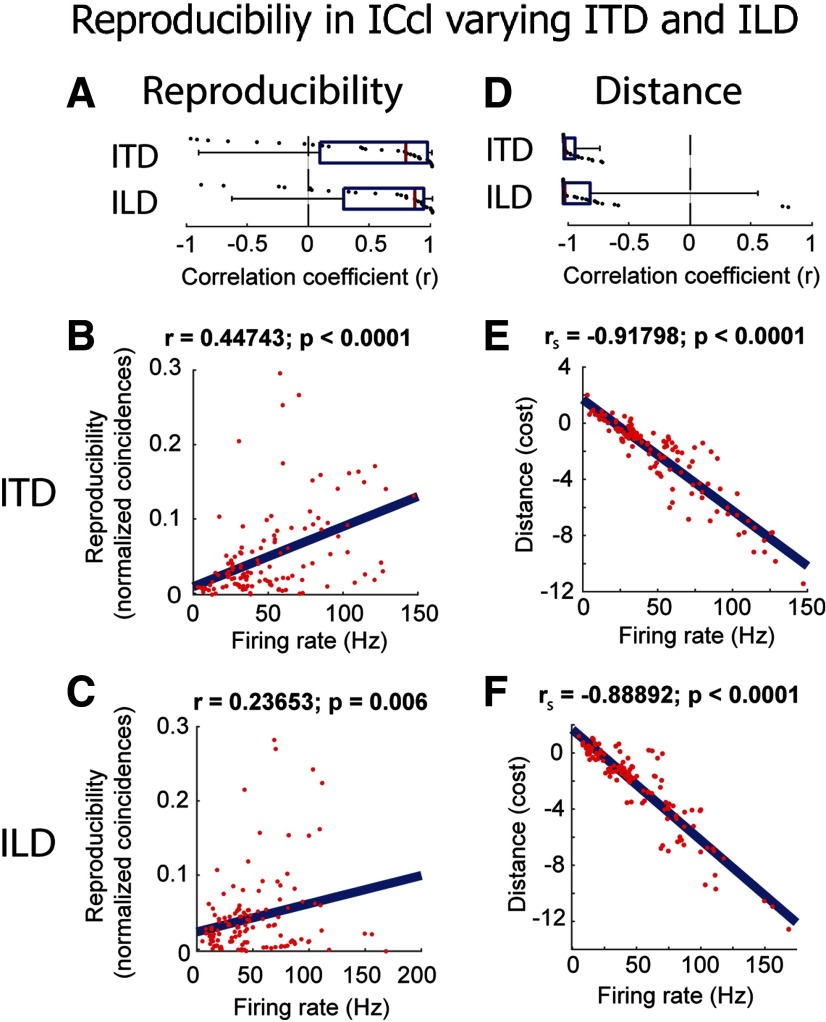
Relationship between reproducibility and firing rate of ICcl neurons (*N* = 33), varying binaural cues. ***A***, Correlation coefficients (*r*) of reproducibility against firing rate in each neuron for varying ITD (top) and ILD (bottom). Red lines indicate the median, blue boxes represent the first and third quartile, and whiskers are the 5th and 95th percentiles. ***B***, ***C***, Reproducibility against firing rate for all neurons for varying ITD (***B***) and ILD (***C***). Red dots represent individual data points and blue lines are linear regressions. Correlation coefficients (*r*) and *p* values are shown above each plot. ***D–F***, Same description as for ***A–C*** but for the corrected Victor–Purpura distance metric.

The reproducibility of the spike patterns was measured at different ITD and ILD values. The relationship between reproducibility and firing rate was assessed with correlation analysis for each individual neuron and across the sample (grouping all points for all neurons). To account for variability across neurons, firing rates and reproducibility were normalized to the maximum for each cell. Reproducibility was significantly correlated with firing rate at varying ITD and ILD. The mean correlation coefficient for individual neurons (*n* = 33) was significantly non-zero (ITD: mean *r* = 0.45 ± 0.63; *t* = 3.68; *p* = 0.0009; two-tailed one-sample *t* test run on *z* values; ILD: mean *r* = 0.57 ± 0.53; *t* = 5.72; *p* < 0.0001; two-tailed one-sample *t* test run on *z* values; Fig. 4*A*). This correlation was also significant after pooling responses across neurons (ITD: *r* = 0.45; *p* < 0.0001; ILD: *r* = 0.24; *p* = 0.006; two-tailed *t* test; Fig. 4*B*,*C*). SACs were normalized by mean firing rate (Joris et al., 2006; Brette, 2012), which accounts for increases in SAC height due to spikes occurring within the same bin due to chance alone. The relationship between response strength and reproducibility indicates an enhancement of patterned spiking evoked by sounds at the neurons’ preferred binaural cues.

As a complementary approach, we assessed the similarity of spiking across trials using a cost-distance metric (Victor and Purpura, 1996). A correction factor was applied to take into account the anticipated effect of increasing numbers of spikes on the distance metric (see Materials and Methods), leading to negative distance values indicating that spike patterns are more similar than what is expected by chance. Across-trial distance of spike trains in ICcl was negatively correlated with firing rate as both ITD and ILD varied. The mean correlation coefficient for individual neurons was significantly non-zero (ITD: mean *r* = −0.94 ± 0.10; *t* = 13.99; *p* < 0.0001; two-tailed one-sample *t* test run on *z* values; ILD: mean *r* = −0.80 ± 0.44; *t* = 8.92; *p* < 0.0001; two-tailed one-sample *t* test run on *z* values; Fig. 4*D*) and the correlation after pooling across neurons was highly significant (Spearman correlation: ITD: *r*_s_ = −0.92; ILD: *r*_s_ = −0.89; both *p* < 0.0001; two-tailed *t* test; Fig. 4*E*,*F*). This negative correlation between across-trial distance and firing rate is consistent with the positive correlation between reproducibility and firing rate reported in Fig. 4 above. These results indicate that the spike-timing reproducibility increases with response strength in ICcl neurons.

**Figure 5.**
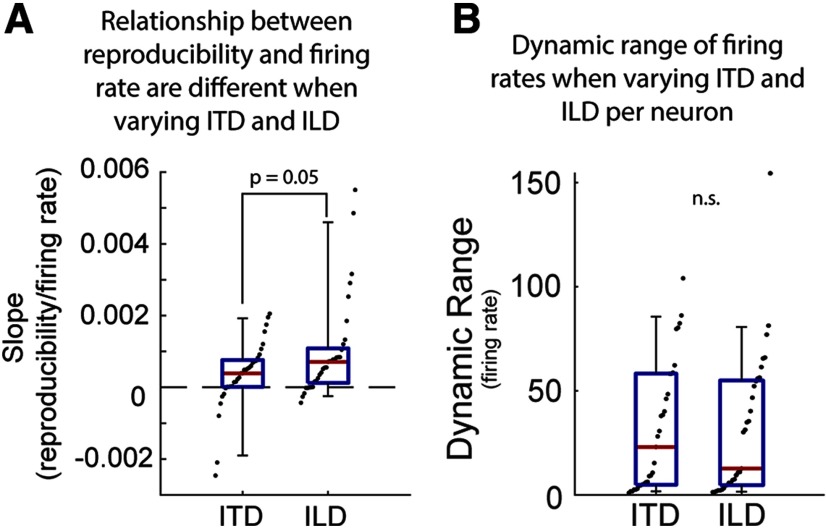
Relationship between reproducibility and response strength depends on the type of binaural cue. ***A***, Comparison of slopes of linear regressions computed for each neuron’s reproducibility as a function of firing rate driven by varying ITD and ILDs. ***B***, Comparison of the dynamic range of firing rates evoked by varying ITD and ILD for each neuron. Red lines indicate the median, blue boxes represent the first and third quartile, and whiskers are the 5th and 95th percentiles.

To assess whether reproducibility could simply be predicted by firing rate, we compared the relationship between reproducibility and firing rate when ITD and ILD were independently changed. For each neuron, a linear regression for reproducibility as a function of firing rate when either ITD or ILD was changed was calculated. We then compared the slopes for each regression. The slopes when ITD was varied (mean slope: 0.0004 ± 0.0009 reproducibility/firing rate units) were moderately shallower than for ILD (mean slope: 0.0010 ± 0.0014 reproducibility/firing rate; *p* = 0.05; two-tailed Wilcoxon signed-rank; Fig. 5*A*). We note that the dynamic range of firing rate elicited by ITD and ILD variations was not significantly different across neurons (*p* = 0.91; two-tailed paired *t* test; Fig. 5*B*), so this difference is not related to the overall number of spikes. This further suggests that while there is a relationship between reproducibility and firing rate for varying ITD and ILD, the rate at which they change may differ and be therefore stimulus-dependent rather than solely rate dependent.

### Stimulus-dependent reproducibility in ICcl for sounds in free-field

**Figure 6.**
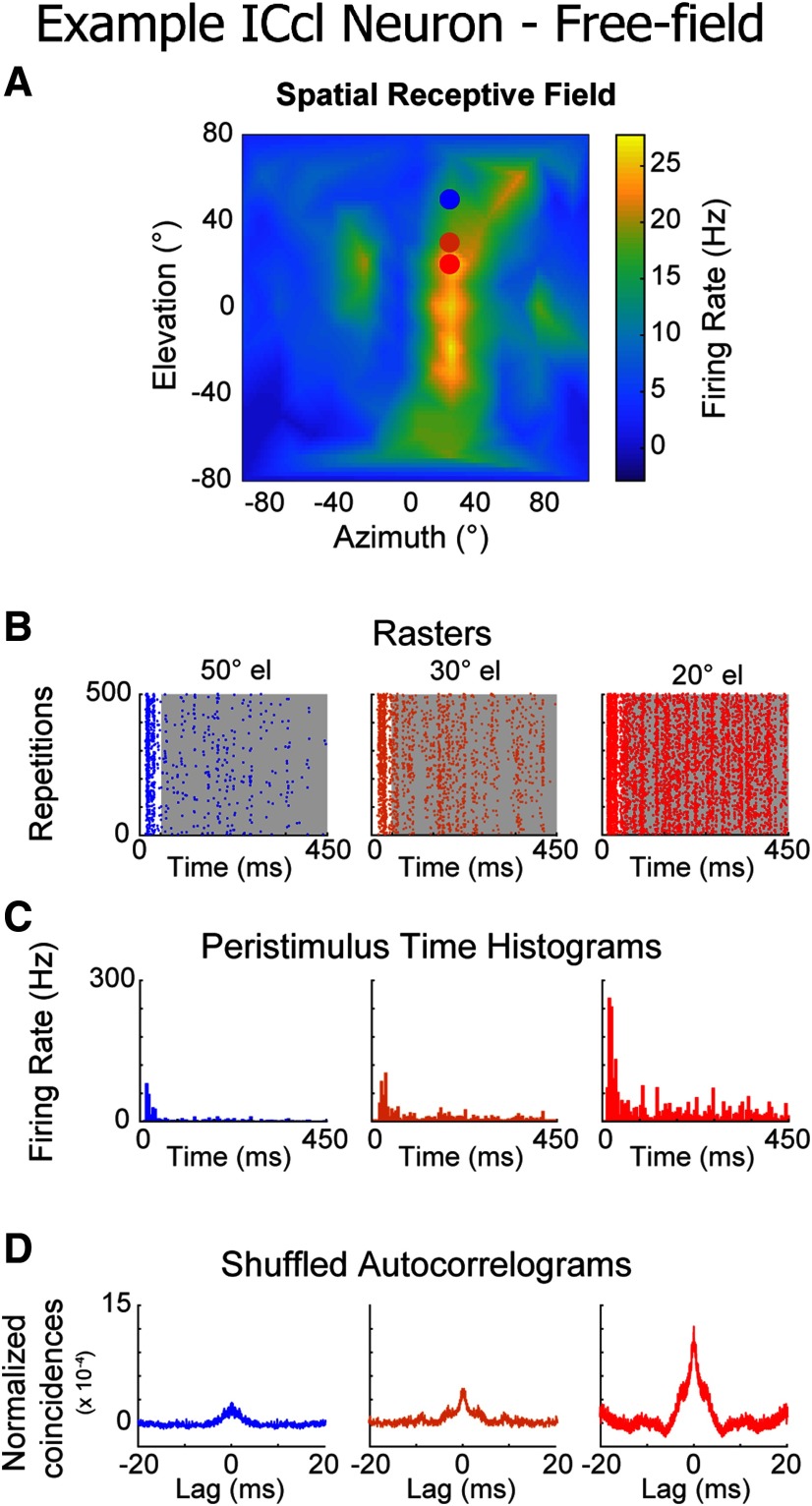
Reproducibility in ICcl to sounds from varying positions in auditory space. ***A***, Example SpRF for an ICcl neuron. Colored dots indicate responses displayed in ***B–D***. ***B***, Spiking raster plots at varying elevations, from the example cell shown in ***A***. Gray background represents region of stimulation used for SAC and distance analysis (50-ms onset responses not considered in the analysis). ***C***, Peristimulus time histograms generated from the raster plots in ***B*** (5-ms bin size). ***D***, SACs generated from the rasters in ***B***.

Dichotic stimulation allows the precise manipulation of individual stimulus parameters, bypassing the filtering properties of the head, described by the head-related transfer function (HRTF). However, these filtering properties modulate sounds in a direction-dependent manner (Keller et al., 1998). Therefore, to test the relationship between sound direction and reproducibility in a more naturalistic setting preserving the natural modulation of the envelope with direction, ICcl responses were also examined using stimuli presented in free-field. ICcl neurons displayed well-delimited spatial tuning (Fig. 6*A*) and reproducible firing patterns for frozen noise in free-field (Fig. 6*B–D*).

**Figure 7.**
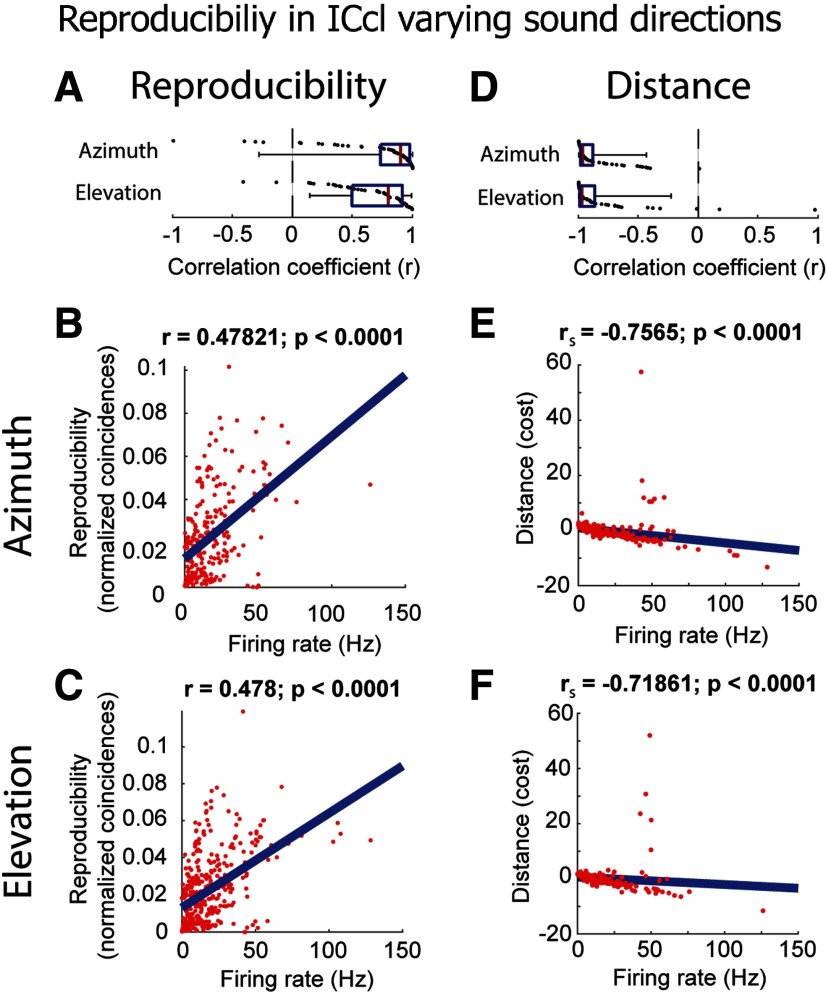
Relationship between reproducibility and firing rate in ICcl (*N* = 64), varying spatial location in free field (same format as in Fig. 4). ***A***, Correlation coefficients (*r*) of reproducibility against firing rate for each neuron for varying azimuth (top) and elevation (bottom). ***B***, ***C***, Reproducibility as a function of firing rate for all neurons for varying azimuth (***B***) and elevation (***C***). Firing rates and reproducibility are normalized by the maximum and minimum for each neuron. ***D–F***, Same description as for ***A–C*** but for the corrected Victor–Purpura distance metric.

Reproducibility was highest at the center of the SpRF and decreased away from the center in both azimuthal and elevational directions, displaying significant positive correlation to firing rate in individual neurons (azimuth: mean *r* = 0.72 ± 0.41; *t* = 9.17; *p* < 0.0001; *n* = 64; two-tailed one-sample *t* test run on *z* values; elevation: mean *r* = 0.70 ± 0.30; *t* = 11.68; *p* < 0.0001; *n* = 64; two-tailed one-sample *t* test run on *z* values; Fig. 7*A*). This relationship held in the pooled data (Pearson correlation: azimuth: *r* = 0.48; elevation: *r* = 0.48; both *p* < 0.0001; two-tailed *t* test; Fig. 7*B*,*C*). Similarly, across-trial distance was negatively correlated with firing rate changes induced by varying sound direction, with significant correlations for individual neurons (azimuth: mean *r* = −0.88 ± 0.19; *p* < 0.0001; *n* = 64; two-tailed Wilcoxon signed-rank test run on *z* values; elevation: mean *r* = −0.85 ± 0.32; *p* < 0.0001; *n* = 64; two-tailed Wilcoxon signed-rank test run on *z* values; Fig. 7*D*) and for pooled data (Spearman correlation: azimuth *r*_s_ = −0.76; elevation *r*_s_ = −0.72; both *p* < 0.0001; two-tailed *t* test; Fig. 7*E*,*F*). These results further demonstrate that reproducibility in ICcl increases with firing rate.

Taken together, the across-trial reproducibility in ICcl was dependent on the firing rate elicited by changes in binaural cues and sound direction. These results show that sound location affects the reproducibility of spike timing across trials in ICcl.

### Reproducibility in OT

We next explored stimulus-dependent reproducibility in the downstream area OT, which contains a map of auditory space (Knudsen, 1982) and commands head-orienting behavior (du Lac and Knudsen, 1990). We recorded deep-layer OT neurons with tetrodes and stimulated with sounds presented in free field, which allowed assessing the population response of local clusters of OT neurons to naturalistic sounds.

Previous reports on neurons in the midbrain map of auditory space showed phase-locking to the sound’s envelope using sinusoidal amplitude-modulated sound, which has a very robust envelope (Keller and Takahashi, 2005; Nelson and Takahashi, 2010). Indeed, tracking such strong amplitude modulations has been hypothesized to be important for differentiating nearby sound sources and suppressing responses to echoes (Keller and Takahashi, 2005; Nelson and Takahashi, 2010; Baxter et al., 2013). However, the across-trial reproducibility and ability to lock to the envelope of stimuli without sinusoidal-amplitude modulations has not been assessed. Typically, neurons lose their ability to lock to lower-order properties of sound (such as phase and envelope) as they progress down the auditory pathways (Johnson, 1980; Winter and Palmer, 1990; Lu et al., 2001; Köppl and Carr, 2003; Joris et al., 2004; Louage et al., 2004; Christianson and Peña, 2006, 2007; Liu et al., 2006; Woolley et al., 2009; Steinberg and Peña, 2011; Kim and Doupe, 2011; Atencio et al., 2012; Calabrese and Woolley, 2015). Therefore, we anticipated that neurons in OT would display a reduced ability to lock to the envelope compared with ICcl neurons.

**Figure 8.**
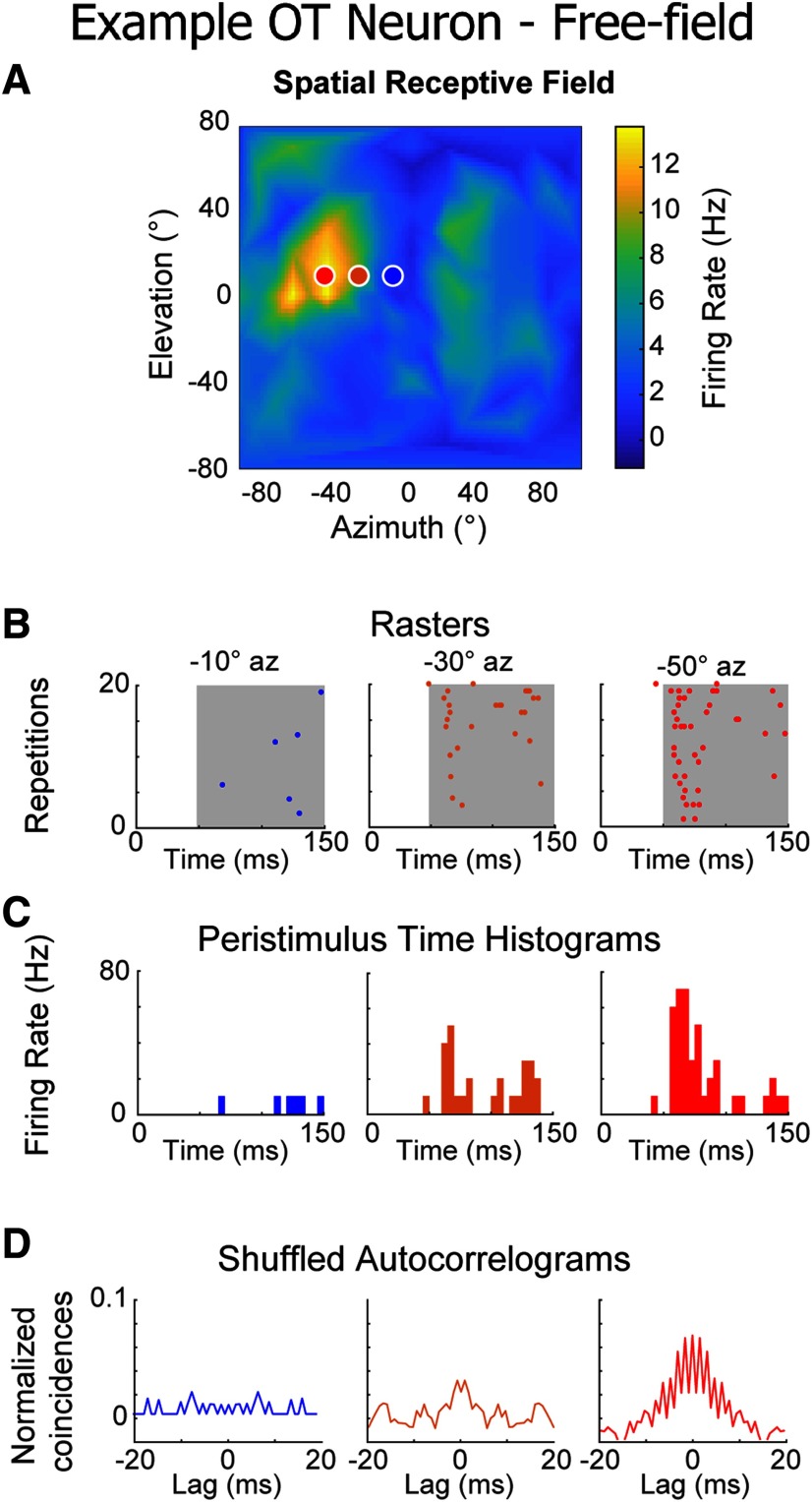
Reproducibility in OT to sounds from varying positions in auditory space. ***A***, Example SpRF of an OT neuron. Colored dots indicate responses displayed in ***B–D***. ***B***, Example spiking raster plots at varying azimuth from the cell shown in ***A***. Gray background represents region of stimulation used for SAC, distance, and synchrony analysis. ***C***, Peristimulus time histograms generated from the raster plots in ***B*** (5-ms bin size). ***D***, SACs generated from the rasters in ***B***.

We examined whether OT neurons displayed reproducible firing patterns in response to frozen noise stimuli in the same manner as in ICcl. In fact, OT neurons (*n* = 93; Fig. 8*A*) showed reproducible spike trains (Fig. 8*B*,*C*), indicated by defined center peaks of their SACs (Fig. 8*D*). However, a substantially larger integration window was required to generate SACs with defined center peaks (Materials and Methods). While the difference in integration windows did not allow a direct comparison of reproducibility values measured by SACs in ICcl and OT neurons, the larger integration window required in OT is consistent with the anticipated decrease of stimulus-induced patterning in this region compared with ICcl. This difference in precision is expected, as envelope-locking generally weakens along auditory pathways (Lu et al., 2001; Joris et al., 2004; Christianson and Peña, 2006, 2007; Steinberg and Peña, 2011). In line with this observation, the distance metric in OT cells was significantly higher than in ICcl (ICcl dichotic: −3.69 ± 3.38, ICcl free-field: −1.27 ± 2.88, OT: 1.16 ± 0.89 cost; Kruskal–Wallis with two-tailed Dunn’s multiple comparison; *p* < 0.0001 across all datasets).

**Figure 9.**
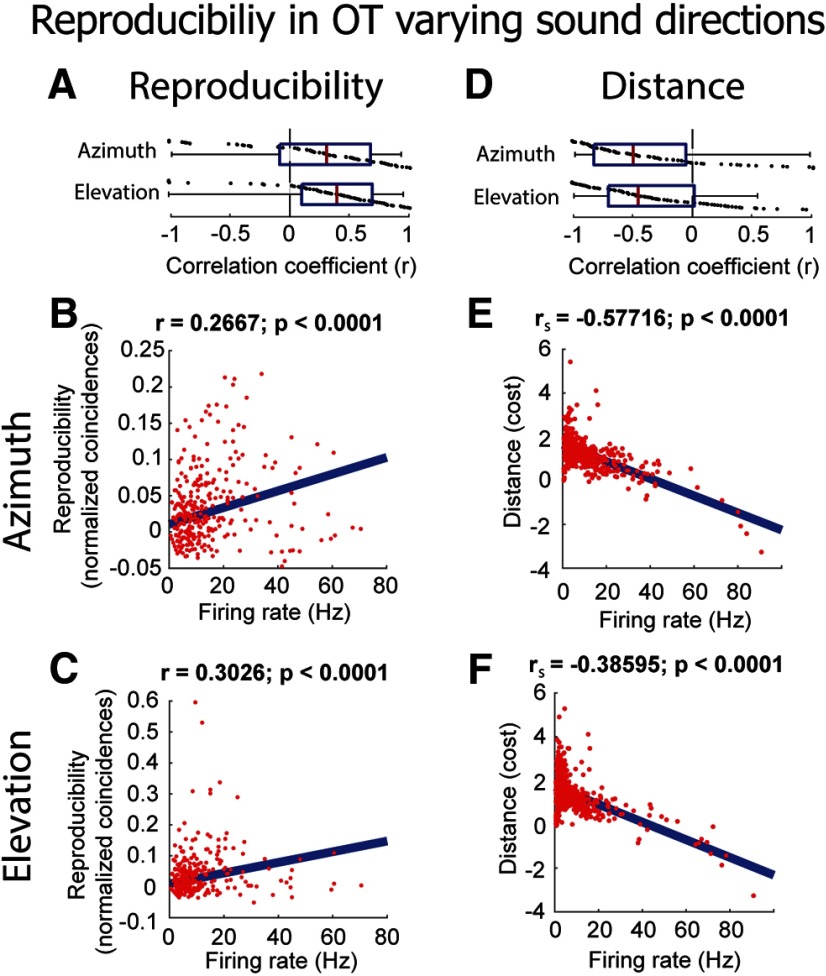
Relationship between reproducibility and firing rate in OT (*N* = 93) varying spatial location (same format as in Fig. 4). ***A***, Correlation coefficients (*r*) of reproducibility against firing rate for each neuron for varying azimuth (top) and elevation (bottom). ***B***, ***C***, Reproducibility as a function of firing rate for all neurons while varying azimuth (***B***) and elevation (***C***). Firing rates and reproducibility are normalized by the maximum and minimum for each neuron. ***D–F***, Same description as for ***A–C*** but for the corrected Victor–Purpura distance metric.

Despite the reduced precision compared with ICcl, reproducibility in OT was still stimulus dependent in individual neurons (azimuth: mean *r* = 0.22 ± 0.57; *p* = 0.0031; *n* = 64; two-tailed Wilcoxon rank-sum test run on *z* values; elevation: mean *r* = 0.29 ± 0.53; *p* < 0.0001; *n* = 64; two-tailed Wilcoxon rank-sum test run on *z* values; Fig. 9*A*) as well as for pooled data points (Pearson correlation: azimuth: *r* = 0.27; elevation: *r* = 0.30; both *p* < 0.0001; two-tailed *t* test; Fig. 9*B*,*C*). Consistently, distance was negatively correlated with response strength to varying sound direction (azimuth: mean *r* = −0.32 ± 0.57; *p* < 0.0001; *n* = 64; two-tailed Wilcoxon rank-sum test run on *z* values; elevation: mean *r* = −0.33 ± 0.49; *p* < 0.0001; *n* = 64; two-tailed Wilcoxon rank-sum test run on *z* values; Fig. 9*D*). This finding indicates that although the precision of spiking in OT is reduced, these neurons produced responses to frozen broadband noise that were more patterned at their preferred direction. This relationship held in the pooled data (Spearman correlation: azimuth: *r*_s_ = −0.58; elevation: *r*_s_ = −0.39; both *p* < 0.0001; two-tailed *t* test; Fig. 9*E*,*F*).

The onset of OT responses was removed from the analysis by calculating reproducibility 50 ms after the start of the stimulus (see Materials and Methods). In addition, to ensure patterning was not driven by the first evoked spike for each neuron, we also repeated the reproducibility analysis with the removal of the first spike after the 50-ms onset window. The correlation between reproducibility and firing rate remained significant for azimuth and elevation both for individual pairs (azimuth: *r* = 0.19 ± 0.64, *p* = 0.03; elevation: *r* = 0.28 ± 0.67, *p* = 0.007; two-tailed Wilcoxon signed-rank run on Z-scores) and across the sampled population (azimuth: *r* = 0.27; elevation: *r* = 0.23; both *p* < 0.0001; two-tailed *t* test). This analysis further validated that results are not significantly affected by the timing of first spikes in OT neurons’ responses, which are characteristically locked to the onset of sound. We note that this further analysis could not be conducted for spike train distance because additional removal of spikes resulted in a substantial increase in the number of trials with absence of spikes, an exclusion criterion for this metric (see Materials and Methods).

Taken together, these results indicate that deep-layer OT neurons display synchronization with the envelope of frozen white noise stimuli at a reduced level compared with ICcl neurons, yet this pattern also varies in a stimulus-dependent manner.

### Stimulus-dependent synchrony in OT

To address the questions whether temporal properties of population responses were stimulus-dependent in the map of space in a manner that could affect coding, spike synchrony of nearby OT neurons was examined. We hypothesized that the stimulus dependent temporal patterns underlying reproducible spiking in ICcl would lead to synchronized responses in similarly tuned ICcl neurons. We further hypothesized that synchronized inputs from ICcl neurons would more efficiently drive spiking in downstream space-map cells, having an impact on their temporal correlations.

**Figure 10.**
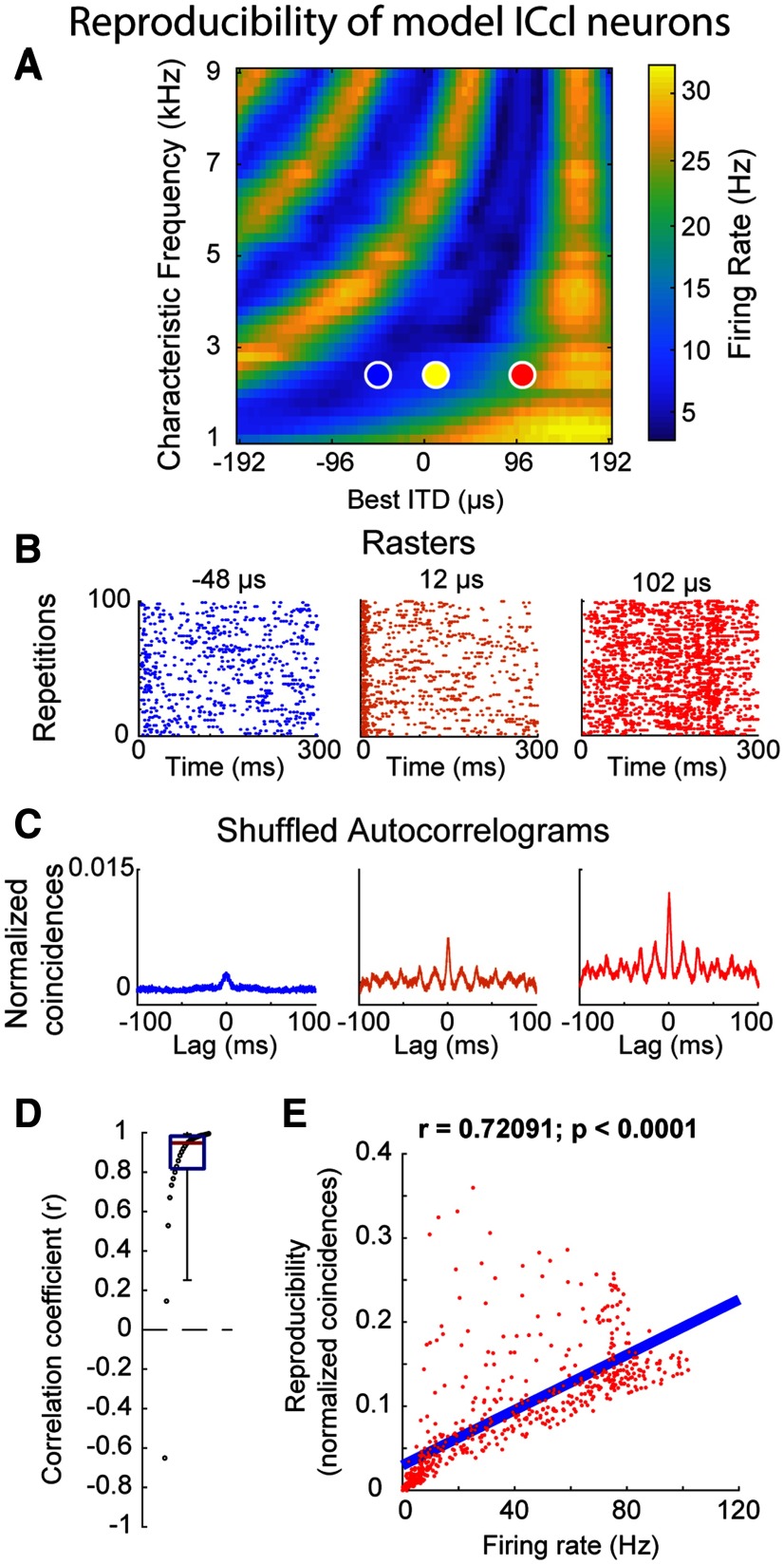
Relationship between reproducibility and firing rate in a modeled ICcl population. ***A***, Heat map of example population response of model ICcl neurons to a sound with 160-μs ITD. Colored dots indicate the example neurons shown in ***B***, ***C***. ***B***, Example rasters of spikes for neurons shown in ***A***. ***C***, Example SACs for neurons shown in ***A***. ***D***, Correlation between reproducibility and firing rate for individual neurons in the population. Box plot shows the median correlation coefficient (red line), first and third quartile (blue box), 5th and 95th percentile (whiskers), and sorted data points. ***E***, Relationship between firing rate and reproducibility for all data points pooled across model neurons. Blue line represents the linear regression. The correlation coefficient (*r*) and exact *p* value are above the plot. Data points in both panels are downsampled (one every 100; 560 out of 55,965 points plotted) to facilitate visualization, while statistical tests and linear regressions were calculated using all values.

These hypotheses were supported by a model of the sound localization pathway. We used a model of the auditory periphery (Fischer et al., 2009) to produce spiking responses of a population of ICcl neurons (Fig. 10*A*). The model included a cochlear filterbank that generated envelope fluctuations in different frequency channels for broadband noise stimuli. The cochlear filterbank outputs on the left and right were cross-correlated to model ITD detection. Gaussian noise was added to the cross-correlation output to model the input to ICcl neurons (Materials and Methods). Model ICcl neurons showed stimulus-dependent reproducibility in the experimentally observed range, both for individual neurons (*r* = 0.85 ± 0.26; two-tailed *t* test; Fig. 10*B–D*) and when data were pooled together (*r* = 0.72; *p* < 0.0001, two-tailed *t* test; Fig. 10*E*). This relationship was expected by the model’s construction, where input signal-to-noise ratio is rate dependent. Interestingly, however, the modeled data displayed asymmetric variability around the regression line of reproducibility as a function of response strength (wider distribution above the regression line), reminiscent of the neural data (Figs. 4, 7).

Spike-time synchrony in the modeled populations was quantified by the integral of the standard-CCG and a shifted-CCG, which accounts for synchrony resulting from similar spike temporal patterns across trials. By construction, the model neurons displayed patterned spiking and thus the shifted-CCG accounted for nearly all synchrony in the standard-CCG, meaning the corrected-CCG (see Materials and Methods) is non-informative in this case.

**Figure 11.**
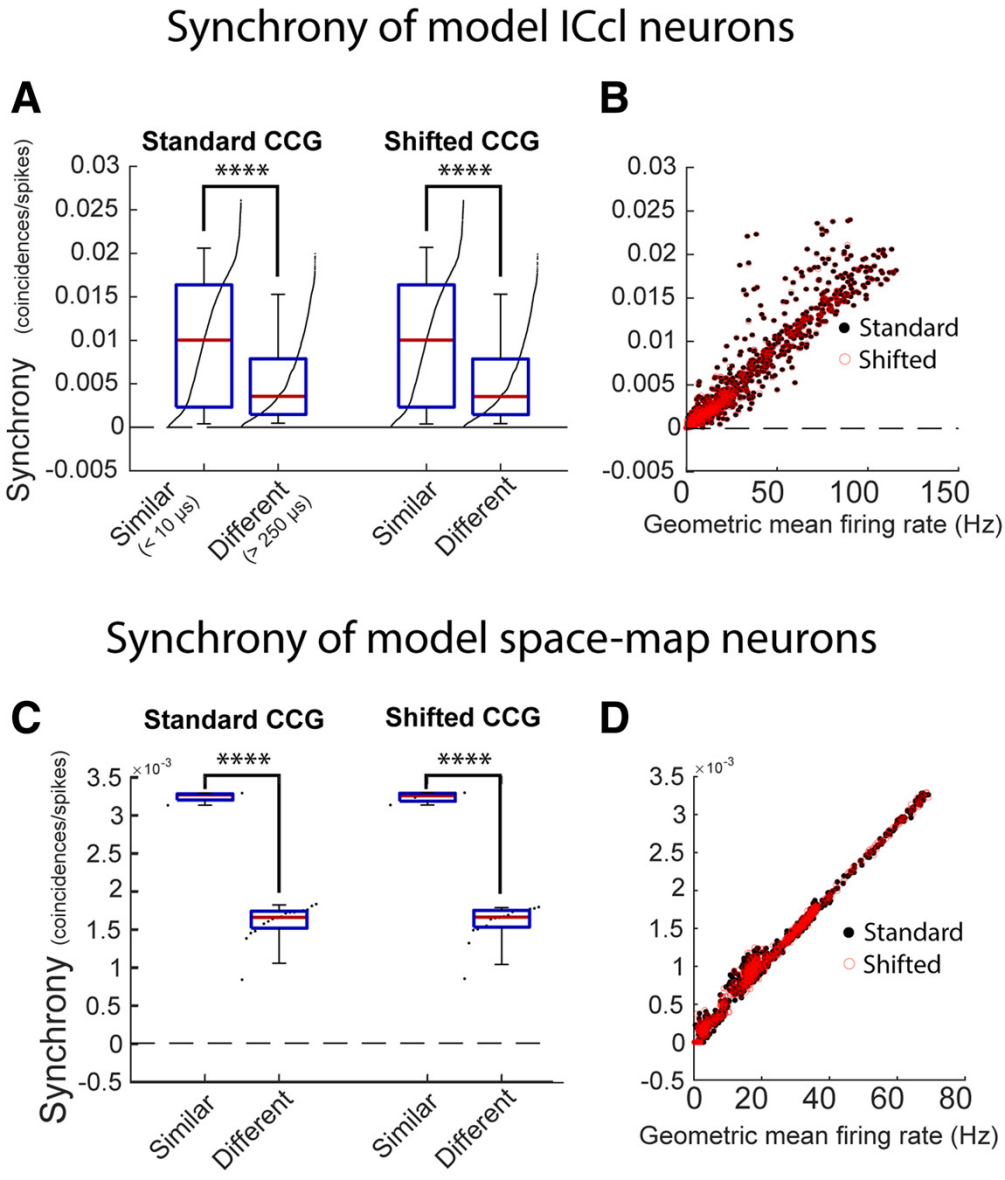
Stimulus-dependent synchrony in the modeled auditory midbrain. ***A***, Comparison of synchrony between neurons of the modeled ICcl population with the same preferred frequency and similar preferred ITD (difference in preferred ITD <10 μs, “similar”) and neurons with the same preferred frequency but different preferred ITDs (>250 μs, “different”), measured by a standard-CCG (left) and a shifted-CCG (right). Definitions for similar and different ITD, specified in the *x*-axis labels for the standard-CCG group in ***A***, are used throughout the figure. ***B***, Relationship between synchrony and response strength (GM firing rate of pairs of neurons) for standard-CCG (black dots) and shifted-CCG (red circles). ***C***, Comparison of synchrony between neurons of the model space-map population tuned to similar ITDs at their peak response (±10 μs from the maximum population response, similar) or between neurons with different preferred ITDs (difference >250 μs, different), measured by a standard-CCG (left) and a shifted-CCG (right). ***D***, Same as ***B*** but for modeled space-map neurons. Box plots show the median synchrony (red line), first and third quartile (blue box), 5th and 95th percentile (whiskers), and raw data points. Data points in all plots are downsampled (one every 100) to facilitate visualization, while statistical tests, linear regression, and histogram were calculated using all values. Mann–Whitney *U* test; ****p < 0.0001.

Supporting the hypotheses, model ICcl neurons with similar preferred ITDs showed stronger spike synchrony than neurons with differing preferred ITDs, quantified by both standard-CCG (*p* < 0.001; two-tailed Mann–Whitney *U*; Fig. 11*A*, left) and shifted-CCG (*p* < 0.001; two-tailed Mann–Whitney *U*; Fig. 11*A*, right). This is further demonstrated by the significant correlation between synchrony and GM firing rate observed for the standard-CCG (*r* = 0.94; two-tailed *t* test; *p* < 0.001) and shifted-CCG (*r* = 0.94; *p* < 0.001; two-tailed *t* test; Fig. 11*B*).

The modeled ICcl neurons formed the input to a model of the midbrain auditory space map. In this map, best frequency is highly correlated with best ITD (Knudsen, 1984; Cazettes et al., 2014). By including this feature into the model, neurons with similar tunings fired synchronously. Figure 11*C* displays a comparison of synchrony assessed by standard-CCG (Fig. 11*C*, left) and shifted-CCG (Fig. 11*C*, right) between the maximally responding unit in a population and neurons with similar ITD tuning (<10-μs preferred ITD) or very different ITD tunings (>250-μs preferred ITD). For both the standard-CCG and shifted-CCG, synchrony between nearby neurons was significantly stronger (both comparisons, *p* < 0.0001; two-tailed Mann–Whitney *U*; Fig. 11*C*), matching the observation in the ICcl input population. Synchrony measured by both the standard-CCG (*r* = 0.995; two-tailed *t* test; *p* < 0.0001) and shifted-CCG (*r* = 0.995; two-tailed *t* test; *p* < 0.0001) was positively correlated to the GM firing rate of the pair (Fig. 11*D*), further demonstrating the stimulus-dependent synchrony. This supports the hypothesis that stimulus dependence underlying reproducible spiking in ICcl would induce synchronized responses in similarly tuned neurons in ICcl and the space map.

**Figure 12.**
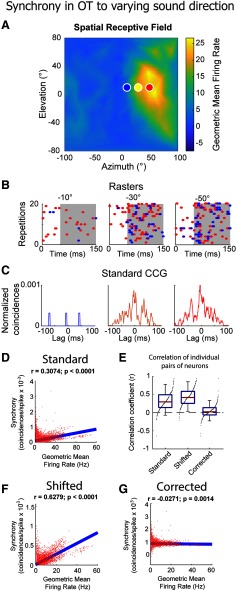
Relationship between synchrony and firing rate in OT, varying location in auditory space. ***A***, Example SpRF computed from the GM firing rate of a pair of OT neurons. Colored dots indicate example locations shown in ***B***, ***C***. ***B***, Example rasters for both neurons (neuron 1 in red and neuron 2 in blue) for the positions in space indicated in ***A*** (analysis window indicated by gray background). ***C***, Example standard-CCGs for locations indicated in ***A***. ***D***, Correlation between synchrony (measured by a standard-CCG) and response strength (GM firing rate) for pooled population data across pairs of neurons. The solid blue line is the linear regression across the entire sample. The correlation coefficient (*r*) and exact *p* value are shown above the plot. ***E***, Distribution of correlation coefficients (*r*) between synchrony assessed by three metrics (standard-CCG, left; shifted-CCG, middle; and corrected-CCG, right) and the GM firing rate for each individual pair of neurons. The red line is the median, the upper and lower bounds of the blue box are the first and third quartile, and whiskers are 5th and 95th percentiles, individual points are all data sorted. ***F***, Correlation between synchrony related to patterning (shifted-CCG) and response strength for pooled population data of all pairs of recorded OT neurons. Same format as in panel ***D***. ***G***, Relationship of the remaining synchrony after subtraction of the shifted-CCG (corrected-CCG) and response strength. Same format as in panel ***D***.

The model’s predictions were tested through *in vivo* recordings of nearby OT with tetrodes. Neurons were stimulated with repeated presentations of identical copies of noise delivered through speakers located at different positions (Materials and Methods). Synchrony and GM firing rate of simultaneously recorded pairs of neurons was quantified using the same methods as for modeled populations (Fig. 12*A–C*). To be included in the analysis, both neurons in a pair were required to display significant response to at least 1 stimulus location (2 SD above mean baseline). This criterion led to five pairs of neurons being omitted; however, all neurons had at least one usable pair (*n* = 194 original pairs, 189 usable pairs). Nearby neurons in OT fired synchronously, as previously reported (Beckert et al., 2017). As predicted by the model, synchrony (assessed by the standard-CCG) was correlated with the GM firing rate both across the sampled population (*r* = 0.31; *p* < 0.0001; two-tailed *t* test; Fig. 12*D*) and in individual pairs of neurons (*r* = 0.31 ± 0.25, *W* = 16 172; *p* < 0.0001; two-tailed Wilcoxon signed-rank run on Z-scores; Fig. 12*E*). We further examined whether stimulus dependent spiking patterns in OT neurons contributed to synchrony by computing a shifted-CCG (Materials and Methods). As observed for the standard-CCG, synchrony quantified by the shifted-CCG was related to the firing rate of the neurons, both for individual pairs (*r* = 0.43 ± 0.24, *W* = 15 061; *p* < 0.0001; two-tailed Wilcoxon signed-rank run on Z-scores; Fig. 12*E*) and across the population (*r* = 0.63; *p* < 0.0001; two-tailed *t* test; Fig. 12*F*). Synchrony quantified by the corrected-CCG, which removes coincidences due to shared patterning (see Materials and Methods), also displayed stimulus-dependency, but at a much weaker level than the standard and shifted-CCGs for individual pairs (*r* = 0.06 ± 0.23, *W* = 7053; *p* = 0.01; two-tailed Wilcoxon signed-rank run on Z-scores; Fig. 12*E*). The correlation between firing rate and synchrony across the population was negative and weak (*r* = −0.036; *p* = 0.0014; two-tailed *t* test; Fig. 12*G*). Together, this further suggests patterning is a fundamental determinant of synchrony.

As for the reproducibility analysis, spike synchrony was also computed excluding the first spike occurring after the removed 50-ms onset window. The correlation between synchrony measured by the standard-CCG and GM firing rate remained significant both for individual pairs (*r* = 0.25 ± 0.35, *p* < 0.0001; two-tailed Wilcoxon signed-rank run on Z-scores) and across the sampled population (*r* = 0.16, *p* < 0.0001; two-tailed *t* test), as well as for the shifted-CCG for individual pairs (*r* = 0.31 ± 0.29, *p* < 0.0001; two-tailed Wilcoxon signed-rank run on Z-scores) and across the population (*r* = 0.46, *p* < 0.0001; two-tailed *t* test). Finally, as observed in the analysis including the first spike, the synchrony not related to patterning, measured by the corrected-CCG, was still stimulus dependent, yet at a weaker level, for individual pairs (*r* = 0.08 ± 0.34, *p* = 0.03; two-tailed Wilcoxon signed-rank run on Z-scores), and negative and weak across the sampled population (*r* = −0.031, *p* = 0.04; two-tailed *t* test). This consistency in the relationship after the removal of the first spike provides additional validation that the correlation between response strength and reproducibility is not an effect of onset responses.

A possible source of synchrony in *in vivo* recordings of anesthetized preparations may be up-and-down states induced by anesthesia (Hahn et al., 2007; Ecker et al., 2014; Poskanzer and Yuste, 2016), where synchronized shifts in firing rate increase correlations (Mochol et al., 2015). To test for this effect, spontaneous activity was recorded in each site and a standard analysis for detecting these states (Pachitariu et al., 2015) was conducted (described in Materials and Methods). Only three out of 33 recordings met the criterion for the presence of up-states using this method (0.7 ± 2.8% of time in up-state). Thus, up-and-down states induced by anesthesia are an unlikely source of synchronization in this dataset. Additionally, midbrain neurons are typically more robust to changes due to anesthesia compared with forebrain neurons (Capsius and Leppelsack, 1996; Gaese and Ostwald, 2001; Syka et al., 2005; Schumacher et al., 2011; Karino et al., 2016). As such, we do not anticipate recordings in awake animals would differ significantly from the present results.

Taken together, synchrony of nearby OT neurons varied in a stimulus-dependent manner, and a substantial portion of this dependency could be explained by changes in the reproducible patterning of responses across trials. We then explored the potential effect of this phenomenon on the readout of the OT neural population using a model population of space-map neurons, presented in the next section below.

### Effect of stimulus-dependent synchrony on the readout of the space-map

**Figure 13.**
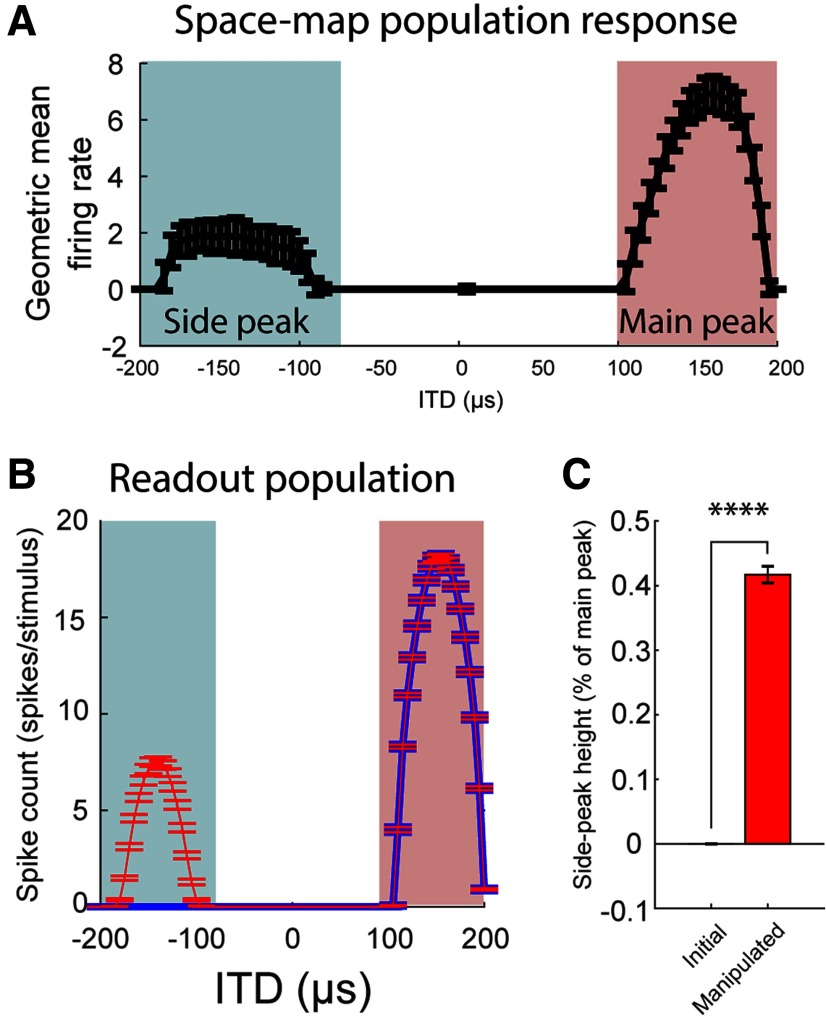
Synchrony dependent readout of a modeled space-map population. ***A***, Average population response of modeled space-map neurons to a sound at 160-μs ITD. Main and side peaks are highlighted in red and blue, respectively. ***B***, Average readout of the space-map from the initial (blue line) and manipulated (red line) modeled populations. Solid lines represent the mean and error bars are the SD. ***C***, Side peak heights relative to the main peak for initial and manipulated modeled populations across all ITDs tested. Paired *t* test, ****p < 0.0001.

To test the effect of stimulus-dependent synchrony on the coding of sound location we modeled space-map neuron populations (Fig. 13*A*) where synchrony in the main and side peaks could either be stimulus dependent or equalized (example population response to a sound of 160-μs ITD shown in Fig. 13*A*). To equalize synchrony in the main peaks and side peaks, we manipulated synchrony in responses to ITDs within the side peaks range by shifting the timing of spikes of the modeled population. As expected, this manipulation increased the synchrony in side peaks relative to the initial population, measured by the standard-CCG (initial = 0.0011 ± 0.00003, manipulated = 0.0014 ± 0.00003 coincidences/spike; *p* < 0.0001, two-tailed *t* test) as well as the shifted-CCG (initial = 0.0011 ± 0.00003, manipulated = 0.0013 ± 0.00003 coincidences/spike; *p* < 0.0001, two-tailed *t* test).

To investigate the effect of spiking synchrony in the space map on the coding of ITD, we modeled the readout of the auditory space map by a downstream population of neurons for both the initial and manipulated populations (Materials and Methods). Consistent with our hypothesis, side peak heights of the space-map population readout were significantly larger when spike times were manipulated to enhance synchrony in side peaks (Fig. 13*B*,*C*; initial = 0.001 ± 0.011%; manipulated = 42 ± 13% main peak; *p* < 0.0001, two-tailed paired *t* test).

Overall, this model shows that side peaks in the downstream output population were larger when synchrony was uniformly high across the population than when synchrony was stimulus dependent, as observed in OT. This suggests that stimulus-dependent synchrony may improve spatial discriminability in the readout of the OT map of space. Taken together, these findings demonstrate a transformation of auditory processing from the central nucleus of the inferior colliculus to the map-of-space where locking to the envelope is degraded while stimulus-dependent co-variability of spiking is preserved in a manner which may have an effect on the coding of sound location.

## Discussion

This study reports stimulus-dependent across-trial reproducibility in the owl’s midbrain nucleus ICcl and the downstream space-map in OT. Although OT neurons displayed lower precision compared with ICcl, synchrony of nearby OT cells was stimulus dependent, consistent with stimulus-dependent reproducibility in ICcl. We further showed that this effect may improve coding of sound location.

### Stimulus-dependent reproducibility in ICcl

Reproducibility in ICcl changed with firing rate as a function of binaural cues and sound direction. Similar stimulus-dependency of reproducibility has been described in mouse retinal ganglion cells (Murphy and Rieke, 2006), cat lateral geniculate nucleus (Cecchi et al., 2000), and rat stellate cells of the cerebellum (Suter and Jaeger, 2004). Thus, this relationship appears to generalize across multiple brain regions. However, we note that while this relationship may be anticipated for many sensory neurons, it is by no means obligatory in auditory neurons. For example, one of the input regions to ICcl, the posterior part of the dorsal lateral lemniscus (LLDp) displays maximal reproducibility when inhibitory input is strongest, not when firing rate is maximal, evidence that temporally precise inhibition can contribute to the reliability of envelope coding (Steinberg et al., 2013). Because the exact mechanism explaining how ILD tuning in ICcl emerges from inhibitory inputs originated in LLDp is still unresolved (Adolphs, 1993), the relationship between the ILD evoking maximal firing rate and reproducibility in ICcl cells and ILD tuning of their inputs from LLDp remains an intriguing question.

Specific mechanisms for stimulus-dependent reproducibility lie with the functional relationship between processes driving firing rate and spectrotemporal tuning of auditory neurons. It has been shown that sharper spectrotemporal tuning increases across-trial reproducibility by enhancing the selectivity of neurons to power fluctuations in the stimulus spectrum (Butts et al., 2007). Previous work has also demonstrated that spectrotemporal tuning of ICcl neurons sharpens with sound level (Keller and Takahashi, 2000). In addition, the computations of ILD and ITD in the brainstem pathways converging on ICcl (Wagner et al., 1987; Takahashi et al., 1989; Adolphs, 1993) are functionally linked to spectrotemporal tuning. As mentioned in the previous section above, in LLDp, where ILD is detected, inhibition modulates spectrotemporal tuning (Steinberg et al., 2013). In parallel, the ITD-detection mechanism in the nucleus laminaris (Carr and Konishi, 1990) is sensitive to time-dependent spectral features of sound (Fischer et al., 2011). Thus, the synchronization of spikes with spectrotemporal features of sounds is expected to change with stimulus parameters (sound level, ITD, and ILD) that also drive the firing rate of ICcl neurons. Our results are consistent with these predictions.

Frozen noise tokens used to assess temporal patterning when manipulating ITD and ILD were randomly varied to avoid potential biases caused by using single signals. The impact of using identical or randomly varying noise tokens is likely small because different examples of broadband white-noise stimuli would not contain large spectrotemporal differences. An interesting test, however, would be to compare reproducibility using the same tokens of naturalistic sounds as ITD and ILD are varied. Further work on this question could link the coding of sound identity and location, building on these initial observations.

Lastly, across stimulus conditions and midbrain areas, the correlation between response strength and the distance metric was stronger than between response strength and reproducibility. A reason for this difference may lie in the formulas used to compute each metric. The distance cost, by definition, cannot be larger than the number of spikes compared, which may result in lower variability when computed for weaker responses. The reproducibility metric, on the other hand, is normalized by firing rate, thus these values will always be between 0 and 1 regardless of number of spikes.

### Dichotic versus free-field stimulation

In this study, we used two complementary stimulation protocols: headphones (dichotic) and free-field stimulation. Stimulus-dependency was observed for both forms of stimulation. Dichotic stimulation permits precise control of the sound signal reaching the eardrum, bypassing confounding factors present in the more naturalistic free-field stimulation, such as the filtering effect of the head. Free-field stimulation is subject to the filtering properties of the head, i.e., the HRTF, which induces co-variability of ITD and ILD (Keller et al., 1998) and variability of binaural cues across frequency and direction (Cazettes et al., 2014, 2016; Fischer and Peña, 2017). Thus, while observing this phenomenon for dichotic stimulation rules out changes in spectrotemporal properties of the sound induced by direction-dependent filtering of the owl’s head and facial ruff as the sole cause of stimulus-dependent reproducibility, the observation that this dependency persisted for sounds in free-field indicates that the underlying mechanism is sufficient to modulate reproducibility in naturalistic conditions for sounds coming from different directions.

### Envelope coding in ICcl

Synchronization with the sound envelope causes reproducible spike-time patterning in ICcl (Keller and Takahashi, 2000). Keller and Takahashi (2000) found no change in spike patterns in seven of eight neurons when sources were placed at different locations that produced similar spike counts, supporting the conclusion that spectrotemporal tuning of these neurons does not qualitatively change with position of the sound source. The present study does not contradict this finding, given it is not stating that the features neurons respond to differ, but only how reliably they respond to them across stimulus locations. Our observations further suggest that envelope coding by ICcl cells is likely dependent on their tuning to spatial cues. It has been hypothesized that locking to the envelope could improve the discrimination between concurrent sound sources at different locations (Keller and Takahashi, 2005) and between acoustic signals and their echoes (Baxter et al., 2013). Stimulus-dependent envelope coding would further strengthen this mechanism, as spiking dependence on the envelope will be reduced for sounds coming from a non-preferred location, making spike patterns of neurons preferring different directions more separable. In the current study, we investigated the effect of stimulus-dependent reproducibility in ICcl on population responses of the downstream OT map of auditory space. However, ICcl also sends projections to the forebrain through the primary auditory thalamus, nucleus ovoidalis (Proctor and Konishi, 1997; Arthur, 2005). The processing of sound may differ in the tectal and thalamic pathways to optimize the coding of sound location and high order spectral features, respectively, in a manner reminiscent of the proposed “where” and “what” pathways (Rauschecker and Tian, 2000; Kuwada et al., 2014). Studies in songbirds have shown a modest impact of sound source location on the coding of song identity by forebrain neurons, but a strong effect of sound location on competing sounds (Maddox et al., 2012). However, these studies were not performed on spatially tuned cells. Further studies can be performed to interrogate downstream effects of stimulus-dependent reproducibility in forebrain regions involved in sound localization.

### Stimulus-dependent reproducibility in OT

Although OT neurons displayed reduced precision compared with ICcl, as anticipated from previous studies of envelope and phase locking across brain regions in the auditory pathway (Johnson, 1980; Winter and Palmer, 1990; Köppl, 1997; Lu et al., 2001; Joris et al., 2004; Louage et al., 2004; Christianson and Peña, 2006, 2007; Liu et al., 2006; Steinberg and Peña, 2011; Yao et al., 2015a,b), their reproducibility was stimulus dependent. This trend is consistent with the notion of stimulus-dependent synchrony in upstream neurons of ICcl driving reproducible firing in OT.

The weaker patterning of recorded (Fig. 8) and modeled (Fig. 10) OT cells relative to ICcl neurons may be due to the broad frequency tuning of space-map neurons that results in a reduction of envelope fluctuations driving OT neurons. Intracellular *in vivo* recordings of ICx cells show strong threshold adaptation (Pena and Konishi, 2002), resulting in fast depolarizations producing spikes with greater fidelity (Platkiewicz and Brette, 2011; Fontaine et al., 2014a,b). Sound stimulation by broadband noise bursts, as used in our study, results in a strong and fast depolarization at the beginning of the stimulus, while during the sustained portion of the stimulus neurons are tonically depolarized, resulting in slower depolarization rates after the onset (Pena and Konishi, 2002). Previous modeling work has demonstrated that slower depolarizations are more vulnerable to noise in the membrane potential (Mainen and Sejnowski, 1995; Berry et al., 1997; Cecchi et al., 2000). Thus, it is expected that spikes occurring after onset responses show less reproducible patterns. OT neurons may also lock to the envelope of stimuli featuring strong correlated modulations, as in sinusoidal-amplitude modulated signals (Keller and Takahashi, 2000, 2005; Nelson and Takahashi, 2010) or complex natural sounds (Escabi and Schreiner, 2002).

### Stimulus-dependent synchrony in OT

Synchrony in OT was stimulus dependent. Our study indicates that a cause of the stimulus-dependent synchrony in OT may be the stimulus-dependent spiking patterns observed in OT and the upstream ICcl. Cochlear filters act as bandpass filters for broadband sounds (Evans, 1989). Because ICcl neurons display narrow frequency tuning, the envelope of their input is expected to strongly fluctuate even for white noise stimuli (Smith, 1997, 2011). Assuming the across-trial reproducibility is a proxy for the precision of spike timing within a given trial, ICcl neurons with the same frequency tuning will undergo strong likelihood of firing more simultaneously as their synchronization with the envelope increases.

The frequency convergence from ICcl into ICx causes these neurons to display frequency tuning within a few kilohertz range (Knudsen and Konishi, 1978a; Knudsen, 1984; Mazer, 1998; Singheiser et al., 2012). Although the broader frequency tuning may attenuate envelope fluctuations under white noise stimulation, ICx neurons remain sensitive to transient depolarizations (Fontaine et al., 2014b). Small amounts of synchronous input among noisy background activity is sufficient to greatly impact spiking efficiency (Rossant et al., 2011). Thus, ICx neurons with similar frequency tuning may display synchronous spiking due to ICcl neurons’ envelope dependent spiking patterns. Mechanistically, this synchrony does not require shared inputs, as the correlations driving the activity exist within the stimulus itself.

At non-preferred sound directions, ICcl neurons will track the sound’s envelope with lower fidelity, resulting in reduced synchrony across frequency channels in ICcl, which will then be relayed downstream to ICx. We therefore hypothesize that binaural cue-dependent synchrony in ICcl underlies binaural cue-dependent synchrony downstream to ICx. Lastly, stimulus-dependent synchrony could be inherited from ICx to OT through the known shared point-to-point projections between these structures (Knudsen and Knudsen, 1983; Beckert et al., 2017). Simultaneous recording of nearby ICcl and ICx cells has proven challenging due to large evoked potentials corrupting spike sorting (Beckert MV and Pena JL unpublished observations). Thus, new techniques will be required to demonstrate that stimulus-dependent synchrony is also observed upstream of OT.

### Effect of stimulus-dependent synchrony on coding

While some studies have considered synchronous spiking detrimental to the coding capacity of a system by reducing temporal sparseness (Itskov and Abbott, 2008; Srivastava et al., 2008; Wiechert et al., 2010), an alternative interpretation is that synchronous firing may be beneficial by increasing the signal-to-noise ratio through temporal summation (Abeles, 1982; Alonso et al., 1996; Usrey et al., 1998; Moreno et al., 2002; Kenyon et al., 2004; Brette, 2012).

Through modeling, we show that side peaks in the population response of the modeled output population were either reduced or completely eliminated when synchrony is stimulus dependent. Assuming that stimulus-dependent synchrony is already present in ICcl, this modeling suggests a mechanism for disambiguating ITD, in addition to the proposed frequency convergence from ICcl to ICx (Takahashi and Konishi, 1986; Trahiotis and Stern, 1989; Mazer, 1998; Peña and Konishi, 2000) for resolving the ambiguity of phase information in the central auditory pathway. Additionally, our study shows that, even if synchrony in OT were a residual effect from its input, a local readout of nearby OT neurons could still enhance accuracy of representing auditory space in an output population. OT neurons are known to project to premotor nuclei that control the owl’s head movement (Masino and Knudsen, 1992, 1993). While the only study of auditory responses of neurons in the motor-associated medial tegmental nucleus that controls the owl’s head movement demonstrates a convergent projection from the midbrain map onto these premotor cells (Cazettes et al., 2018), this study also showed that removing side peaks was a critical aspect of reading out OT’s population to produce observed premotor tegmentum responses, further supporting the functional relevance of stimulus-dependent synchrony in OT for coding auditory space.

## Conclusions

We have demonstrated that the across-trial precision of spike timing of neurons in the barn owl midbrain is stimulus dependent, increasing at the neurons’ preferred direction. Though this patterning dissipates in the downstream map-of-space, a stimulus-dependent synchrony is observed. Modeling shows that this stimulus dependent synchrony may have functional implications for the coding of auditory space, thus suggesting a functional interaction between the tuning to spectrotemporal features and to sound location in auditory neurons. Stimulus-dependent reproducibility and synchrony are consistent with multiple cellular and network mechanisms affecting spiking precision proposed in other brain regions (Mainen and Sejnowski, 1995; Berry et al., 1997; Berry and Meister, 1998; Cecchi et al., 2000; Butts et al., 2007), suggesting these findings are generalizable across neurons sensitive to the temporal structure of the stimulus.

## Synthesis

Reviewing Editor: Tatyana Sharpee, The Salk Institute for Biological Studies Decisions are customarily a result of the Reviewing Editor and the peer reviewers coming together and discussing their recommendations until a consensus is reached. When revisions are invited, a fact-based synthesis statement explaining their decision and outlining what is needed to prepare a revision will be listed below. The following reviewer(s) agreed to reveal their identity: Go Ashida.

Neurons along the sensory pathways respond to repeated presentations of an identical stimulus in a highly reproducible manner. How this response reproducibility is related to neuronal coding, however, remains largely uncovered. The present paper studies this question in barn owls’ auditory midbrain, where a topographic representation (the so-called auditory space map) of the external acoustic world is formed. In vivo physiological recordings in combination with computational modeling revealed that the stimulus-dependent reproducibility of the spiking activity of midbrain neurons depends not only on their mean spiking rate but also on their tuning properties (i.e., preferred direction of the sound stimulus). Overall, the paper is well written, but a number of additional analyses are needed to demonstrate that 1) the increased reproducibility is not simply due to the increased rate and 2) that the optic tectum results are not simply based on the onset response. Finally, both reviewers questioned the relevance of the model in guiding the interpretation of neural data. In particular, whether the reproducibility values of the model neurons are similar to the physiological data (most figures showed data normalized to the minimum and maximum per cell).

Both reviewers question the appropriateness of the term “entrain/entrainment” here. In the context of auditory neuroscience, entrainment (of spiking activity) usually indicates that there is one-to-one correspondence between the spike timing and reference (stimulus) cycles (e.g., Joris et al., 1994, J Neurophysiol). In the present study, however, the authors used broadband noise, which has no clear periodicity. Therefore, the term “entrainment” might not be appropriate. The authors cite several references when they mention entrainment. However, none of these references call reproducible spiking entrainment. The suggestion is to revise the manuscript without using the term “entrain/entrainment”, except where the authors clearly refer to periodic stimuli (such as sinusoidally amplitude-modulated sounds). Alternative wording would be “stimulus-dependent reproducibility”, “patterned activity”, “across-trial temporal fidelity”, etc.

Reviewer 1.

*** General Comments ***

The downstream neuron model (Fig 12) did not unequivocally demonstrated that “stimulus-dependent synchrony is expected to improve spatial discriminability in the readout of the OT map of space” (lines 554-555). Fig 12 shows that the side band would increase when the input spikes are manipulated, but not that the side band is reduced when stimulus-dependent synchrony exists. Furthermore, OT neurons project to motor-associated nuclei that control the head movement (e.g., Masino & Knudsen, 1992, Exp Brain Res; Masino & Knudsen, 1993, J Neurosci). If these downstream neurons actually need to obtain improved spatial discriminability; their functions might be different. The suggestion is to revise the corresponding text in the section “Effect of stimulus-dependent synchrony on the readout of the space-map” (page 24), preferably mentioning the functions of the downstream neurons with citations.

*** Specific Points ***

- Lines 26-27: “stimulus-dependent entrainment predicts stimulus-dependent synchrony” is unclear to me, since “entrainment” and “synchrony” are not defined. You should rephrase this sentence.

- Lines 47-48: Here you could mention the across-frequency integration in the ICx.

- Line 61 “Reproducibility in ICcl was correlated with firing rate changes”: Was it correlated with “rate changes”, not with “rate” itself?

- Line 84: How many owls did you use?

- Lines 162-163: According to the text, the SAC peak was normalized by bin size, number of trials, and firing rate, while the equation here shows the normalization only with the mean rate. Please revise the equation according to the correct description.

- Line 180: “reproducibility values were normalized to their range”: What do you mean by “their range” here? Is it the range for each neuron, for the entire pooled population, or some other defined range? Please explain.

- Line 188: Please specify the unit for Delta t (and the factor q).

- Line 192: “optimal (lowest cost) solution” of what? Please explain whose cost function was minimized and how this minimized solution was used for your further analyses.

- Line 219-222: Is x the spike count or spike rate? Please explain. If it is the spike rate, you will need a unit.

- Line 233: Please add a comma after “synchrony”.

- Lines 237-238 “how patterned responses influenced synchrony”: Do you mean “how patterned response in each neuron influenced synchrony across neurons”? Please clarify.

- Line 247: Please add a comma after “in space”.

- Line 269: Please add “and” after the comma.

- Line 291: You need to add a unit (mV) for Delta=1.

- Line 296: What is the unit for the parameter a? And what is the unit for si(t)? I suppose they should have the unit for conductance, so that the equation gi(t) = si(t) + ni(t) will make sense.

- Line 296: “s(t)” should be “si(t)”?

- Line 297: You should either remove the word “weighted” or add a weight parameter in “gi(t) = si(t) + ni(t)”.

- Liens 302-303: I would suggest using a different subscript for the parameters ki and Vi, since they do not depend on the unit number i.

- Line 308: Please explain what N denotes and what value you used for it.

- Line 309: Did you use the same parameter values (Delta, VL, tau, etc.) as for the ICcl model? Please explain.

- Lines 311-313: This sentence is unclear to me. What were the variances of these Gaussians? How did you define the difference between ONE central Gaussian and the TWO other Gaussians? If necessary, making a small schematic figure might help.

- Line 321: Again, please explain what N denotes and what value you used for it.

- Line 328: “the suppression of side-peaks” of what? Perhaps of CGG?

- Line 363: I would not call the curve in Fig 3A (right) sigmoidal, as the spike rate monotonically decreases with ILDs over 10 dB. Please reword.

- Line 423: “sinusoidal-amplitude modulated” should be “sinusoidal amplitude-modulated” (move the hyphen).

- Line 452: “white noise” should be “broadband noise”.

- Line 486: Here you could refer back to your Figs 7-8 and discuss that the across-frequency integration between ICcl and OT might be the reason for the reduced reproducibility in OT.

- Lines 516-517 “The amount of synchrony once coincidences due to patterning were removed...”: This is an awkward sentence. Please rephrase.

- Line 541: “side peaks the” should be “side peaks of the”?

- Line 617: To be consistent with the lines 615-616, [“what” and “where”] should be [“where” and “what”].

- Lines 677-682: As noted in my general comments, I would like to see some more careful discussions on the downstream stages.

- Lines 631-632: Here you could cite your Fig 10, which showed reduced reproducibility between units having different frequency tunings.

- Line 712: Please add the article number.

- Line 740: “Du Lac” should be “du Lac”.

- Line 794: Please remove the question mark.

- Line 821: Please add the journal name and pages.

- Line 914: “pars” should be “part”.

- Legend of Fig 2: How many spike trains did you generate?

- Legend of Fig 3A: What was the value of ILD for the ITD tuning curve? And what was the value of ITD for the ILD tuning curve?

- Legend of Fig 3C: What was the bin size for the PSTHs?

- Legend of Fig 4B-F: How many neurons are used for the plots?

- Legend of Fig 4B-F: What are the red dots? Do they correspond to the grayscale on the background? Please explain.

- Legend of Figs 6 and 8: You could save the space by simply saying “Same format as in Fig 4”.

- Legend of Fig 10: How many data points did you have and how many points are shown?

- Legend of Fig 11C-D: You could remove the redundancy by saying “Same format as in panel A”.

- Figure 9: Showing only the summary statistics is not very informative. I would add ITD and ILD tuning curves, raster plots, and SACs of the modeled ICcl neuron (as in Fig 3), so that the readers could see how well the model replicates the corresponding data.

- Figure 11A,C,D: These panels are too busy to understand. I would suggest dividing each panel into two: one showing the bar graphs and one showing the grayscale and regression lines.

- Figure 12A: I would suggest adding the text “main peak” and “side peak” to the panel.

Reviewer 2:

The authors present electrophysiological data from ICcl neurons and OT neurons in response to broadband sound stimuli that vary in ITD/ILD or location in the anesthetized owl. They calculate reproducibility of temporal patterns in spiking across trials and find that in ICcl as well as in OT, reproducibility increases with spike rate, which the authors interpret as stimulus-dependent increases in reproducibility. They then measured synchrony between nearby OT neurons using tetrodes, predicting that the rate-dependent reproducibility of ICcl neurons would result in rate-dependent synchrony in OT. They used a computational model suggesting that stimulus-dependent synchrony can improve spatial coding.

The amount of data and techniques used in the paper is impressive. The positive correlation between dependence of reproducibility in ICcl and spike rate is interesting, new and different than in other nuclei such as LLDp (Steinberg and Pena, 2013). However, the claim that reproducibility is thus stimulus-dependent needs to be further substantiated. There are major concerns regarding interpretation of the OT data: the evidence for the claim that these neurons have significant reproducibility and correlations that can improve spatial coding at the population level, is not sufficiently convincing in the manuscript.

Reviewer 2:

Major comments:

1. The authors find a positive correlation between reproducibility and spike rate in ICcl neurons, and link this to changes in binaural cues, but to make that last claim (e.g. L685-686), the data is underanalyzed: authors only indirectly show the effect of binaural cues on reproducibility through spike rate. How does reproducibility vary as a function of ITD/ILD, and how does this relation relate to rate-ITD/ILD functions? For different ITDs or ILDs that result in similar spike rates, is reproducibility also similar? How does reproducibility vary with absolute rate (cannot be seen in the current figures, due to normalization, see comment 5 below)? Is there a flooring/ceiling effect for low/high spike rates?

2. One of the main points in this paper is that the OT neuron firing patterns are locked to stimulus waveforms which causes repetitive temporal firing patterns across trials and peaked CCGs between pairs of neurons. However, the evidence for this claim in the paper is not convincing.

2.1 For the example neuron in Fig. 7, response onset only occurs at ∼50 ms from stimulus onset, thus within the analysis window used to calculate the SAC, and therefore the small SAC peak may be mainly driven by onset effects. This casts doubt on the interpretation of the population data in Fig.8 and in Fig. 11. Steinberg and Pena (2011) discard the first 100 ms when calculating SACs, to make sure the neuron is in steady state. What does the reproducibility analysis (for individual examples and across the population) look like when the first 100 ms is discarded? From the data presented in the manuscript it is not convincing that there are reproducible temporal firing patterns across trials for OT neurons, in stark contrast to the ICcl neuron in Fig. 3.

2.2 The same problem may thus also be true for the CCG data from OT. How does Fig. 11 look like when 100 ms instead of 50 ms is used as definition of onset response? The authors should also show some example neurons here in addition to the population analysis.

3. L. 250-254 says that responses from different stimuli were grouped before calculating CCGs. This analysis therefore cannot support the conclusion from the authors that “spike time synchrony between nearby OT neurons was highest for sounds at their preferred direction”, since responses from different directions are mixed together.

4. L. 18 ‘...inherent to the sounds identity...’: there is no evidence for this in this paper. The authors do not present data comparing neuronal responses to different stimuli.

5. Figure 4 panels B-F and similar panels in other figures: it is not very informative to normalize reproducibility, a metric that is already normalized, and rate for each cell separately:

a) because of this within-neuron-normalization before calculating an overall correlation, this analysis ‘after pooling across neurons’ is basically the same as Fig. 4 panel A

b) this way the reader cannot compare the data between ICcl and OT, and to published normalized SACs (as in Louage et al 2004,2005; Steinberg and Pena 2011). It is better to (also) report normalized SAC peak values as in these papers. Steinberg and Pena (2013) report reliability actually as the normalized peak of the SAC.

c) the resulting panels are highly skewed, which is visible from the heat maps where typically two black squares are visible at two opposite corners. The distribution of the red dots is thus not very informative as most of them are stacked on top of each other over the black heat map squares. I recommend changing the display so that the reader can appreciate the range of actual (normalized) SAC as well as the range of spike rates. Different individual data points belonging to the same neuron can then be connected with lines.

6. The authors should compare reproducibility values of the physiological data from ICcl and OT neurons with respectively the model ICcl neurons and the model space map neurons. E.g. in Fig. 10A,C, synchrony of model ICcl neurons and space-map neurons are similar in value. This is not expected based on the physiology, where reproducibility seems way lower for OT than for ICcl.

7. The model allows the authors to do an additional analysis, similar to Fig 10 B/D, to show that it is really the stimulus waveform that matters rather than firing rate: they could compare synchrony separately for pairs of model neurons where ITD is the same vs. different, but GM firing rate is the same. This could show that the synchrony present in ICcl can be an additional source of information for space-map neurons to improve their ITD tuning.

8. Fig 11: similar as for Fig. 4B-F: synchrony should not be normalized to minimum and maximum per cell. It is already a dimensionless metric and the absolute values should be shown, to be able to estimate the amount of synchrony in OT and compare to ICcl and the model.

9. The rationale of the section starting at L537 is not very compelling to me: the authors seem to suggest here that the (likely limited) synchrony present in OT neurons may be able to influence downstream neurons. Is it not much more likely that the clear synchrony in ICcl neurons affects OT neurons, and that the small amount of synchrony remaining in the OT population is just a ‘left-over’ without having any additional function?

10. Also for that same analysis: for the interpretation it is critical that the amount of synchrony in the modeled space-map neurons is physiologically realistic, i.e. similar as in the recorded OT neurons. This is not clear.

11. Throughout the manuscript the term ‘entrainment’ is used, but it is confusing here and should be avoided in this paper. In auditory neurophysiology entrainment has a very specific meaning, i.e. a firing pattern to tones where a spike is fired in every stimulus cycle (discussed for example in Joris and Smith, Neuroscience 2008). This is not how this term is used here. For the non-periodic stimuli used here entrainment is not defined.

Minor comments:

• L130: Describe more fully the characteristics of the broadband sound: shape of amplitude and phase spectrum? Frozen noise? Same token only within session or throughout the experiment?

• L139-141: same remark as for L130

• L150: duration of time windows used to measure spontaneous activity?

• L156-158: best to describe stimuli fully, once in the Methods. See remark L130.

• L160-163: Please describe more precisely the normalization of SAC and calculation of reproducibility. This is an important point as the relation between reliability and spike rate is one of the main findings of the paper. This will allow the reader to compare the results more easily to other physiological works that have used normalized SACs (Louage et al., 2004,2005; Steinberg and Pena 2011; Steinberg and Pena 2013), where SAC was normalized by dividing by N(N-1)r2ΔtD (N: number of trials; r: spike rate; Δt: bin width; D: stim duration). As mentioned earlier, it would be less confusing to report normalized SAC peak values, i.e. reliability as used in Steinberg et al. (2013)

• L169: can the authors comment on the choice to integrate only over the half-width of the peak as opposed to (Brette, 2012)? How would the results be different if the full width was used?

• L188: the units of Delta-t need to be given in order to make sense of the values of q. q should be set such that the spike train distance and SAC are computed with the same resolution (as in Steinberg and Pena, 2011)

• L241: why is for CCG the full peak used in the integral as opposed to the halfwidth for SAC? (see L169)

• L480: ‘shift-corrected’: same as shift-predictor?

• L 517: please be more precise how this corrected CCG is calculated.

• L 541: missing word between ‘peaks’ and ‘the’

• L573: please explain why in ICcl, reproducibility would increase with spike rate, whereas in LLDp, an input to ICcl, this is not the case?

• L688-690: this sentence does not make much sense to me.

• Fig. 3D: wrong Y axis label: not reproducibility is shown, but complete SACs.

• Legend Fig. 3: please specify the convention used for positive/negative ITDs and ILDs.

• Fig. 4B,C and similar panels: Y axis label ‘coincidences/spikes’ is not correct here. This is reproducibility, normalized for each cell to its minimum/maximum, so the resulting number cannot be interpreted any more as ‘coincidences/spikes’

• Fig. 9: Position of numbers on x and y axes seems wrong, as some datapoints have values >1.

• Fig. 10 panel A: ‘shifted CCG’. Please be consistent throughout the manuscript, either ‘shifted CCF’ or ‘shift predictor CCG’.

• Legend/Fig 12: please be more explicit throughout that these are all results from modeled neurons

## Author Response

Dear Dr Sharpee,

We thank you and reviewers for the very thoughtful and constructive suggestions. We have done our best to address them. Below is a point-by-point response to editor’s and reviewers’ comments.

Sincerely,

The Authors

Synthesis Statement for Author (Required):

Neurons along the sensory pathways respond to repeated presentations of an identical stimulus in a highly reproducible manner. How this response reproducibility is related to neuronal coding, however, remains largely uncovered. The present paper studies this question in barn owls’ auditory midbrain, where a topographic representation (the so-called auditory space map) of the external acoustic world is formed. In vivo physiological recordings in combination with computational modeling revealed that the stimulus-dependent reproducibility of the spiking activity of midbrain neurons depends not only on their mean spiking rate but also on their tuning properties (i.e., preferred direction of the sound stimulus). Overall, the paper is well written, but a number of additional analyses are needed to demonstrate that 1) the increased reproducibility is not simply due to the increased rate and 2) that the optic tectum results are not simply based on the onset response.

Response: We thank the editor for the clear summary of requested changes. We have conducted further analysis and added a new section to the results titled “Increased reproducibility in ICcl is not simply due to increased firing rate", a new figure and a paragraph describing this analysis in the methods section, reporting that responses with similar firing rate but driven by different stimulus parameters were not predictive of spiking reproducibility. We have also revised the description of the methods and figures to clarify the fact that onset responses were excluded from the analysis of reproducibility in optic tectum responses.

Finally, both reviewers questioned the relevance of the model in guiding the interpretation of neural data. In particular, whether the reproducibility values of the model neurons are similar to the physiological data (most figures showed data normalized to the minimum and maximum per cell).

Response: The model was calibrated to yield firing rates and reproducibility values that are consistent with the data. We agree that this cannot be assessed from our normalized plots. We now show un-normalized values in a new Figure10.

Both reviewers question the appropriateness of the term “entrain/entrainment” here. In the context of auditory neuroscience, entrainment (of spiking activity) usually indicates that there is one-to-one correspondence between the spike timing and reference (stimulus) cycles (e.g., Joris et al., 1994, J Neurophysiol). In the present study, however, the authors used broadband noise, which has no clear periodicity. Therefore, the term “entrainment” might not be appropriate. The authors cite several references when they mention entrainment. However, none of these references call reproducible spiking entrainment. The suggestion is to revise the manuscript without using the term “entrain/entrainment", except where the authors clearly refer to periodic stimuli (such as sinusoidally amplitude-modulated sounds). Alternative wording would be “stimulus-dependent reproducibility", “patterned activity", “across-trial temporal fidelity", etc.

Response: We thank reviewers very much for noticing this incorrect terminology and for suggesting alternative wording. We have revised the manuscript now referring to patterned activity and stimulus-dependent reproducibility. The term ‘entrainment’ is only used once in the manuscript when referring to previous work on sinusoidally amplitude-modulated sounds.

Reviewer 1.

*** General Comments ***

The downstream neuron model (Fig 12) did not unequivocally demonstrated that “stimulus-dependent synchrony is expected to improve spatial discriminability in the readout of the OT map of space” (lines 554-555). Fig 12 shows that the side band would increase when the input spikes are manipulated, but not that the side band is reduced when stimulus-dependent synchrony exists.

Response: We have rewritten the text describing the model to clarify the implications of the model. The manipulation in Figure 12 (now Figure 13) shows that if synchrony was uniformly high across the population, then the side peaks would be larger. This shows that the presence of stimulus-dependent synchrony of the type observed creates smaller side peaks (we understand this is what the reviewer meant by ‘side band’) than what would exist in the uniformly high synchrony condition.

Furthermore, OT neurons project to motor-associated nuclei that control the head movement (e.g., Masino & Knudsen, 1992, Exp Brain Res; Masino & Knudsen, 1993, J Neurosci). If these downstream neurons actually need to obtain improved spatial discriminability; their functions might be different. The suggestion is to revise the corresponding text in the section “Effect of stimulus-dependent synchrony on the readout of the space-map” (page 24), preferably mentioning the functions of the downstream neurons with citations.

Response: To address this issue we have revised the last section of the discussion, titled “Effect of stimulus-dependent synchrony on coding", adding the following text: “OT neurons are known to project to premotor nuclei that control the owl’s head movement (Masino and Knudsen, 1992; 1993). While the only study of auditory responses of neurons in the motor-associated medial tegmental nucleus that controls the owl’s head movement demonstrates a convergent projection from the midbrain map onto these premotor cells (Cazettes et al. 2018, this study also showed that removing side peaks was a critical aspect of reading out OT’s population to produce observed premotor tegmentum responses, further supporting the functional relevance of stimulus-dependent synchrony in OT for coding auditory space.”

*** Specific Points ***

Response: We thank Reviewer 1 very much for the attention and effort put in the suggested edits and corrections.

- Lines 26-27: “stimulus-dependent entrainment predicts stimulus-dependent synchrony” is unclear to me, since “entrainment” and “synchrony” are not defined. You should rephrase this sentence.

Response: This sentence was rephrased as follows: “Theoretical analysis showed that stimulus-dependent temporal patterning predicts stimulus-dependent synchrony in nearby cells sharing input with similar spectrotemporal structure, which in turn can sharpen the downstream readout of sound direction.”

- Lines 47-48: Here you could mention the across-frequency integration in the ICx.

Response: This sentence was revised to: “ICx neurons receive convergent input from ICcl (Wagner et al., 1987), which sharpens their spatial tuning through frequency convergence (Takahashi and Konishi, 1986; Mazer, 1995; Peña and Konishi, 2000).”

- Line 61 “Reproducibility in ICcl was correlated with firing rate changes”: Was it correlated with “rate changes”, not with “rate” itself?

Response: Thank you for catching this confusing wording. We removed ‘changes’, from the beginning of this sentence which now reads as follows: “Reproducibility in ICcl was correlated with firing rate, as ITD and ILD...”

- Line 84: How many owls did you use?

Response: We have added a specification in the methods section on how many owls were used in this study (5 owls: 4 male, 1 female).

- Lines 162-163: According to the text, the SAC peak was normalized by bin size, number of trials, and firing rate, while the equation here shows the normalization only with the mean rate. Please revise the equation according to the correct description.

Response: Thank you for pointing out this error in statement of the reproducibility formula. Bin size was corrected for because we estimated the integral of the SAC peak but it is not directly in the formula. Additionally, firing rate is calculated by average response strength, which is how number of trials are controlled for. We have corrected the formula to just normalizing by firing rate.

- Line 180: “reproducibility values were normalized to their range”: What do you mean by “their range” here? Is it the range for each neuron, for the entire pooled population, or some other defined range? Please explain.

Response: This text was removed because normalized values are no longer reported.

- Line 188: Please specify the unit for Delta t (and the factor q).

Response: We have updated the methods to state that delta t is in seconds and the scaling factor q is unitless.

- Line 192: “optimal (lowest cost) solution” of what? Please explain whose cost function was minimized and how this minimized solution was used for your further analyses.

Response: We apologize for this confusion. We computed all possible permutations of spike-train adjustments (spike-time shift, spike removal, or added spikes) and selected the solution which has the smallest cost value. We have updated the text to reflect this more explicitly, as follows: “Permutations of all possible elementary transformations were performed using MatLab code (code obtained from the laboratory website of Dr. Jonathan Victor at Cornell Medical School: http://www-users.med.cornell.edu/∼jdvicto/pubalgor.html), the solution which displayed the smallest final cost was then selected and this cost was reported.”

- Line 219-222: Is x the spike count or spike rate? Please explain. If it is the spike rate, you will need a unit.

Response: Thank you for noticing this missing unit. We have updated the text to explain x is geometric mean firing rate (spikes/s).

- Line 233: Please add a comma after “synchrony”.

Response: Comma added as suggested.

- Lines 237-238 “how patterned responses influenced synchrony”: Do you mean “how patterned response in each neuron influenced synchrony across neurons”? Please clarify.

Response: We revised this sentence to improve clarity as follows: “Here we used this CCG to investigate whether synchrony of nearby cells could be influenced by shared properties of the patterned activity of these cells.”

- Line 247: Please add a comma after “in space”.

Response: Revised as suggested.

- Line 269: Please add “and” after the comma.

Response: Revised as suggested.

- Line 291: You need to add a unit (mV) for Delta=1.

Response: Revised as suggested.

- Line 296: What is the unit for the parameter a? And what is the unit for si(t)? I suppose they should have the unit for conductance, so that the equation gi(t) = si(t) + ni(t) will make sense.

Response: The parameter a is unitless, as are s_i(t) and g_i(t). In this form of the membrane equation, g_i(t) represents the input conductance, divided by the constant leak conductance. We now state this in the methods.

- Line 296: “s(t)” should be “si(t)”?

Response: Revised as suggested.

- Line 297: You should either remove the word “weighted” or add a weight parameter in “gi(t) = si(t) + ni(t)”.

Response: Thank you for catching this. We removed the word weighted.

- Lines 302-303: I would suggest using a different subscript for the parameters ki and Vi, since they do not depend on the unit number i.

Response: Thank you for catching this. We revised the subscripts to avoid confusion with the neuron index.

- Line 308: Please explain what N denotes and what value you used for it.

Response: We revised the text to state that N = 2665 is the number of input ICcl neurons.

- Line 309: Did you use the same parameter values (Delta, VL, tau, etc.) as for the ICcl model? Please explain.

Response: We now explain that the parameters are the same as for the ICcl model.

- Lines 311-313: This sentence is unclear to me. What were the variances of these Gaussians? How did you define the difference between ONE central Gaussian and the TWO other Gaussians? If necessary, making a small schematic figure might help.

Response: The pattern of the weights is created by one central Gaussian that represents an excitatory center from which two Gaussians, one on either side of the center, are subtracted to represent an inhibitory surround. We now include an equation to illustrate the form of the weights and list the variances of the Gaussians.

- Line 321: Again, please explain what N denotes and what value you used for it.

Response: We revised the text to state that N = 81 is the number of input space-map neurons.

- Line 328: “the suppression of side-peaks” of what? Perhaps of CGG?

Response: We now state that we are measuring suppression of side-peaks forming in the pattern of spike counts produced by the output population when plotted as a function of the preferred ITD

- Line 363: I would not call the curve in Fig 3A (right) sigmoidal, as the spike rate monotonically decreases with ILDs over 10 dB. Please reword.

Response: The end of this sentence was reworded as follows: “...and rate-ILD curves displayed sigmoidal or open peak shapes favoring contralateral side louder (Figure 3A).”

- Line 423: “sinusoidal-amplitude modulated” should be “sinusoidal amplitude-modulated” (move the hyphen).

Response: Corrected as suggested.

- Line 452: “white noise” should be “broadband noise”.

Response: Revised as suggested.

- Line 486: Here you could refer back to your Figs 7-8 and discuss that the across-frequency integration between ICcl and OT might be the reason for the reduced reproducibility in OT.

Response: We are grateful for this constructive suggestion but unfortunately could not understand what exactly the reviewer meant. Line 486 was part of a paragraph referring specifically to modeled ICcl neurons.

- Lines 516-517 “The amount of synchrony once coincidences due to patterning were removed...”: This is an awkward sentence. Please rephrase.

Response: The description and denomination of cross-correlograms (CCGs) was extensively edited for clarification. Specifically, the beginning of this sentence was rephrased as follows: “Synchrony quantified by the corrected CCG, which removes coincidences due to shared patterning (see methods), also displayed stimulus-dependency,...”

- Line 541: “side peaks the” should be “side peaks of the”?

Response: Revised as indicated.

- Line 617: To be consistent with the lines 615-616, [“what” and “where”] should be [“where” and “what”].

Response: Thank you; revised as suggested.

- Lines 677-682: As noted in my general comments, I would like to see some more careful discussions on the downstream stages.

Response: As indicated above, the last section of the discussion was revised to address this issue.

- Lines 631-632: Here you could cite your Fig 10, which showed reduced reproducibility between units having different frequency tunings.

Response: We thank the reviewer for this suggestion. This sentence was revised to include the now Figure 11, as follows: “The weaker patterning of recorded (Figure 8) and modeled OT cells relative to ICcl neurons (Figure 11) may be due to the broad frequency tuning of space-map neurons that results in a reduction of envelope fluctuations driving OT neurons.”

- Line 712: Please add the article number.

Response: Link to this eNeuro paper added.

- Line 740: “Du Lac” should be “du Lac”.

Response: Revised as indicated.

- Line 794: Please remove the question mark.

Response: Typo corrected.

- Line 821: Please add the journal name and pages.

Response: Thank you for detecting this typo. Mazer 1995 was an error. We removed this citation and replaced it by Mazer 1998 (PNAS).

- Line 914: “pars” should be “part”.

Response: Revised as indicated.

- Legend of Fig 2: How many spike trains did you generate?

Response: The legend was revised, stating that “A total of 20,000 simulations were run.”

- Legend of Fig 3A: What was the value of ILD for the ITD tuning curve? And what was the value of ITD for the ILD tuning curve?

Response: We have specified these values in the figure. Thank you.

- Legend of Fig 3C: What was the bin size for the PSTHs?

Response: Bin size (5ms) is now reported in the legend.

- Legend of Fig 4B-F: How many neurons are used for the plots?

Response: We have added N’s in each figure legend (4, 7, and 9) to specify how many neurons were used in each figure.

- Legend of Fig 4B-F: What are the red dots? Do they correspond to the grayscale on the background? Please explain.

Response: We have revised the figure legend, clarifying that red dots are individual datapoints and have removed grayscale for all plots.

- Legend of Figs 6 and 8: You could save the space by simply saying “Same format as in Fig 4”.

Response: To address the reviewer’s request, we added “(same format as in Fig 4)” after the title of Figs 7 and 9, and removed the description of the plots redundant with Figure 4.

- Legend of Fig 10: How many data points did you have and how many points are shown?

Response: The legend was revised, adding that 560 out of 55965 points were plotted.

- Legend of Fig 11C-D: You could remove the redundancy by saying “Same format as in panel A”.

Response: Revised as suggested.

- Figure 9: Showing only the summary statistics is not very informative. I would add ITD and ILD tuning curves, raster plots, and SACs of the modeled ICcl neuron (as in Fig 3), so that the readers could see how well the model replicates the corresponding data.

Response: We have expanded this figure (now figure 10) to include examples as suggested.

- Figure 11A,C,D: These panels are too busy to understand. I would suggest dividing each panel into two: one showing the bar graphs and one showing the grayscale and regression lines.

Response: We have simplified the plots by removing the grayscale and binning. We are now reporting non-normalized data and a single regression line.

- Figure 12A: I would suggest adding the text “main peak” and “side peak” to the panel.

Response: Text has been added as suggested.

Reviewer 2:

The authors present electrophysiological data from ICcl neurons and OT neurons in response to broadband sound stimuli that vary in ITD/ILD or location in the anesthetized owl. They calculate reproducibility of temporal patterns in spiking across trials and find that in ICcl as well as in OT, reproducibility increases with spike rate, which the authors interpret as stimulus-dependent increases in reproducibility. They then measured synchrony between nearby OT neurons using tetrodes, predicting that the rate-dependent reproducibility of ICcl neurons would result in rate-dependent synchrony in OT. They used a computational model suggesting that stimulus-dependent synchrony can improve spatial coding.

The amount of data and techniques used in the paper is impressive. The positive correlation between dependence of reproducibility in ICcl and spike rate is interesting, new and different than in other nuclei such as LLDp (Steinberg and Pena, 2013). However, the claim that reproducibility is thus stimulus-dependent needs to be further substantiated. There are major concerns regarding interpretation of the OT data: the evidence for the claim that these neurons have significant reproducibility and correlations that can improve spatial coding at the population level, is not sufficiently convincing in the manuscript.

Reviewer 2:

Major comments:

1. The authors find a positive correlation between reproducibility and spike rate in ICcl neurons, and link this to changes in binaural cues, but to make that last claim (e.g. L685-686), the data is underanalyzed: authors only indirectly show the effect of binaural cues on reproducibility through spike rate. How does reproducibility vary as a function of ITD/ILD, and how does this relation relate to rate-ITD/ILD functions? For different ITDs or ILDs that result in similar spike rates, is reproducibility also similar? How does reproducibility vary with absolute rate (cannot be seen in the current figures, due to normalization, see comment 5 below)? Is there a flooring/ceiling effect for low/high spike rates?

Response: We thank the reviewer for raising this important question. As indicated above, we conducted further analysis and added a new section to the results titled “Increased reproducibility in ICcl is not simply due to increased firing rate” and a new figure, reporting that responses with similar firing rate but driven by different stimulus parameters were not predictive of reproducibility. In addition, all normalized plots have been remade plotting non-normalized data.

2. One of the main points in this paper is that the OT neuron firing patterns are locked to stimulus waveforms which causes repetitive temporal firing patterns across trials and peaked CCGs between pairs of neurons. However, the evidence for this claim in the paper is not convincing.

2.1 For the example neuron in Fig. 7, response onset only occurs at ∼50 ms from stimulus onset, thus within the analysis window used to calculate the SAC, and therefore the small SAC peak may be mainly driven by onset effects. This casts doubt on the interpretation of the population data in Fig.8 and in Fig. 11. Steinberg and Pena (2011) discard the first 100 ms when calculating SACs, to make sure the neuron is in steady state. What does the reproducibility analysis (for individual examples and across the population) look like when the first 100 ms is discarded? From the data presented in the manuscript it is not convincing that there are reproducible temporal firing patterns across trials for OT neurons, in stark contrast to the ICcl neuron in Fig. 3.

Response: We have clarified in the text that onset responses of OT neurons were omitted from all analyses.

2.2 The same problem may thus also be true for the CCG data from OT. How does Fig. 11 look like when 100 ms instead of 50 ms is used as definition of onset response? The authors should also show some example neurons here in addition to the population analysis.

Response: As indicated in the response to the previous (2.1) comment, the onset component of OT responses was removed from all data analysis. We would also like to note that OT responses have a more distinct and transient onset than the cochlear nucleus neurons studied by Steinberg and Pena (2011). Because of this difference, we judged that removing the initial 50ms was sufficient to do away with the onset component of OT responses. Removing the first 100ms may also affect the statistical power of our data by decreasing the number of spikes used for the analysis, because OT neurons do not display high firing rates. Additionally, we have included example plots to supplement the population analysis as suggested.

3. L. 250-254 says that responses from different stimuli were grouped before calculating CCGs. This analysis therefore cannot support the conclusion from the authors that “spike time synchrony between nearby OT neurons was highest for sounds at their preferred direction", since responses from different directions are mixed together.

Response: We no longer bin the CCGs and are reporting non-normalized data in the figures and results.

4. L. 18 ‘...inherent to the sounds identity...’: there is no evidence for this in this paper. The authors do not present data comparing neuronal responses to different stimuli.

Response: The last sentence of the abstract was revised using more parsimonious language, as follows: “This study reports a functional connection between spike patterning elicited by spectrotemporal features of a sound and the coding of its location.”

5. Figure 4 panels B-F and similar panels in other figures: it is not very informative to normalize reproducibility, a metric that is already normalized, and rate for each cell separately:

a) because of this within-neuron-normalization before calculating an overall correlation, this analysis ‘ after pooling across neurons’ is basically the same as Fig. 4 panel A

b) this way the reader cannot compare the data between ICcl and OT, and to published normalized SACs (as in Louage et al 2004,2005; Steinberg and Pena 2011). It is better to (also) report normalized SAC peak values as in these papers. Steinberg and Pena (2013) report reliability actually as the normalized peak of the SAC.

c) the resulting panels are highly skewed, which is visible from the heat maps where typically two black squares are visible at two opposite corners. The distribution of the red dots is thus not very informative as most of them are stacked on top of each other over the black heat map squares. I recommend changing the display so that the reader can appreciate the range of actual (normalized) SAC as well as the range of spike rates. Different individual data points belonging to the same neuron can then be connected with lines.

Response: As suggested by the reviewer, we have rerun the analysis and remade plots to remove this added layer of normalization.

6. The authors should compare reproducibility values of the physiological data from ICcl and OT neurons with respectively the model ICcl neurons and the model space map neurons. E.g. in Fig. 10A,C, synchrony of model ICcl neurons and space-map neurons are similar in value. This is not expected based on the physiology, where reproducibility seems way lower for OT than for ICcl.

Response: We have adjusted the model parameters so reproducibility (for ICcl) and synchrony (for OT) are within the same order of magnitude as the model. In particular we have modified the parameters representing: 1) signal-to-noise ratio (SNR) for the ICcl and ICx neurons, 2) the bandwidth of the frequency convergence from the ICcl to ICx neurons, 3) the weight of modeled ICx and output population inputs, and 4) the membrane potential formulas.

7. The model allows the authors to do an additional analysis, similar to Fig 10 B/D, to show that it is really the stimulus waveform that matters rather than firing rate: they could compare synchrony separately for pairs of model neurons where ITD is the same vs. different, but GM firing rate is the same. This could show that the synchrony present in ICcl can be an additional source of information for space-map neurons to improve their ITD tuning.

Response: We thank the reviewer for this interesting suggestion. Figure 11 compares synchrony for similar and different ITDs.

8. Fig 11: similar as for Fig. 4B-F: synchrony should not be normalized to minimum and maximum per cell. It is already a dimensionless metric and the absolute values should be shown, to be able to estimate the amount of synchrony in OT and compare to ICcl and the model.

Response: Normalization has been removed as suggested.

9. The rationale of the section starting at L537 is not very compelling to me: the authors seem to suggest here that the (likely limited) synchrony present in OT neurons may be able to influence downstream neurons. Is it not much more likely that the clear synchrony in ICcl neurons affects OT neurons, and that the small amount of synchrony remaining in the OT population is just a ‘left-over’ without having any additional function?

Response: We thank the reviewer for this thoughtful question. To try addressing this issue, we added the following text in the discussion section titled “Effect of stimulus-dependent synchrony on coding”: “Through modeling, we show that side-peaks in the population response of the modeled output population were either reduced or completely eliminated when synchrony is stimulus-dependent. Assuming that stimulus-dependent synchrony is already present in ICcl, this modeling suggests an additional mechanism for disambiguating ITD to the proposed frequency convergence from ICcl to ICx (Takahashi and Konishi, 1986; Trahiotis and Stern, 1989; Mazer, 1998; Peña and Konishi, 2000) for resolving the ambiguity of phase information in the central auditory pathway. Additionally, our study shows that, even if synchrony in OT were a residual effect from its input, a local readout of nearby OT neurons could still enhance accuracy representing auditory space in an output population.”

10. Also for that same analysis: for the interpretation it is critical that the amount of synchrony in the modeled space-map neurons is physiologically realistic, i.e. similar as in the recorded OT neurons. This is not clear.

Response: Synchrony is now at the same order of magnitude for the model and OT recordings.

11. Throughout the manuscript the term ‘entrainment’ is used, but it is confusing here and should be avoided in this paper. In auditory neurophysiology entrainment has a very specific meaning, i.e. a firing pattern to tones where a spike is fired in every stimulus cycle (discussed for example in Joris and Smith, Neuroscience 2008). This is not how this term is used here. For the non-periodic stimuli used here entrainment is not defined.

Response: We thank both reviewers for this very useful advice. As mentioned above, the terminology has been revised, no longer using ‘entrainment’.

Minor comments:

L130: Describe more fully the characteristics of the broadband sound: shape of amplitude and phase spectrum? Frozen noise? Same token only within session or throughout the experiment?

Response: We have clarified that a new frozen noise token was generated for each neuron in the “Response reproducibility” section, and that for ITD and ILD characterization the stimuli were not frozen.

L139-141: same remark as for L130

Response: Same edits were done for this section.

L150: duration of time windows used to measure spontaneous activity?

Response: We have clarified that we counted the spontaneous activity from an equivalent amount of time to the stimulus duration preceding the stimulus onset.

L156-158: best to describe stimuli fully, once in the Methods. See remark L130.

Response: Description of the stimuli has been expanded in the above sections and removed from this section as suggested.

L160-163: Please describe more precisely the normalization of SAC and calculation of reproducibility. This is an important point as the relation between reliability and spike rate is one of the main findings of the paper. This will allow the reader to compare the results more easily to other physiological works that have used normalized SACs (Louage et al., 2004,2005; Steinberg and Pena 2011; Steinberg and Pena 2013), where SAC was normalized by dividing by N(N-1)r2ΔtD (N: number of trials; r: spike rate; Δt: bin width; D: stim duration). As mentioned earlier, it would be less confusing to report normalized SAC peak values, i.e. reliability as used in Steinberg et al. (2013)

Response: We have expanded upon the normalization procedure in lines with methodology proposed by Brette (2012) and are also now reporting the reproducibility values without the extra normalization as explained above.

L169: can the authors comment on the choice to integrate only over the half-width of the peak as opposed to (Brette, 2012)? How would the results be different if the full width was used?

Response: We used half-width to omit areas of SACs that in our data that were more prone to variation, and less informative about the reproducibility of patterning.

L188: the units of Delta-t need to be given in order to make sense of the values of q. q should be set such that the spike train distance and SAC are computed with the same resolution (as in Steinberg and Pena, 2011)

Response: We have added the units as suggested. The selection of q does match our SAC resolution used for ICcl. We do note that changing q does not change the overall relationship for distance.

L241: why is for CCG the full peak used in the integral as opposed to the halfwidth for SAC? (see L169)

Response: We wished to capture as many spiking coincidences as possible considering the lower firing rate in OT, and using a narrower window did not change the overall trends we are reporting (relationship between response strength and synchrony is still present).

L480: ‘shift-corrected’: same as shift-predictor?

Response: We agree with the reviewer the standard terminology used for CCGs is confusing. We have edited the entire manuscript to make the terms for the CCGs more consistent and understandable. For this particular line, the answer is ‘no’, this is what remains after the shift-predictor (now referred to simply as “shifted CCG”) is subtracted.

L 517: please be more precise how this corrected CCG is calculated.

Response: Additional text has been added, as follows: “To evaluate coincidences not related to temporal dynamics of the stimulus, the shifted-CCG was subtracted from the standard-CCG for each neuron (coincidences for each time lag bin in the shifted-CCG were removed from the matching bin in standard-CCG), to produce a corrected-CCG.”

L 541: missing word between ‘peaks’ and ‘the’

Response: typo corrected, adding ‘of’ between ‘peaks’ and ‘the’

L573: please explain why in ICcl, reproducibility would increase with spike rate, whereas in LLDp, an input to ICcl, this is not the case?

Response: We thank the reviewer for this interesting question, which unfortunately remains open. To address this issue we revised the first paragraph of the discussion section titled “Stimulus-dependent reproducibility in ICcl” as follows: “Reproducibility in ICcl changed with firing rate as a function of binaural cues and sound direction. Similar stimulus-dependency of reproducibility has been described in mouse retinal ganglion cells (Murphy and Rieke, 2006), cat lateral geniculate nucleus (Cecchi et al., 2000), and rat stellate cells of the cerebellum (Suter and Jaeger, 2004). Thus, this relationship appears to generalize across multiple brain regions. However, we note that while this relationship may be anticipated for many sensory neurons, it is by no means obligatory in auditory neurons. For example, one of the input regions to ICcl, the posterior pars of the dorsal lateral lemniscus (LLDp) displays maximal reproducibility when inhibitory input is strongest, not when firing rate is maximal, evidence that temporally precise inhibition can contribute to the reliability of envelope coding (Steinberg et al., 2013). Because the exact mechanism how ILD tuning in ICcl emerges from inhibitory inputs originated in LLDp is still unresolved (Adolphs 1993), the relationship between the ILD evoking maximal firing rate and reproducibility in ICcl cells and ILD tuning of their inputs from LLDp remains an intriguing question.”

L688-690: this sentence does not make much sense to me.

Response: To try making a clearer and more parsimonious statement, we revised this sentence as follows: “Modeling shows that this stimulus dependent synchrony may have functional implications for the coding of auditory space, thus suggesting a functional interaction between the tuning to spectrotemporal features and to sound location in auditory neurons.”

Fig. 3D: wrong Y axis label: not reproducibility is shown, but complete SACs.

Response: We have corrected to just say “normalized coincidences"

Legend Fig. 3: please specify the convention used for positive/negative ITDs and ILDs.

Response: The legend was revised adding “Positive ITDs and ILDs indicate, respectively, sounds leading and louder on the right ear.”

Fig. 4B,C and similar panels: Y axis label ‘coincidences/spikes’ is not correct here. This is reproducibility, normalized for each cell to its minimum/maximum, so the resulting number cannot be interpreted any more as ‘coincidences/spikes’

Response: We have corrected the axes to “normalized coincidences"

Fig. 9: Position of numbers on x and y axes seems wrong, as some datapoints have values >1.

Response: Now displaying raw values.

Fig. 10 panel A: ‘shifted CCG’. Please be consistent throughout the manuscript, either ‘shifted CCF’ or ‘shift predictor CCG’.

Response: The description of each type of cross-correlogram was revised for clarity in the methods and denominations made uniform across the manuscript.

Legend/Fig 12: please be more explicit throughout that these are all results from modeled neurons.

Response: The term ‘modeled’ was included in the description of every panel of the figure.
